# Private haplotypes can reveal local adaptation

**DOI:** 10.1186/1471-2156-15-61

**Published:** 2014-05-22

**Authors:** Agnès E Sjöstrand, Per Sjödin, Mattias Jakobsson

**Affiliations:** 1 Department of Evolutionary Biology, Evolutionary Biology Centre, Uppsala University, Uppsala, Sweden; 2 UMR 7206 Eco-anthropologie et Ethnobiologie, CNRS-MNHN-Université Paris VII, Paris, France; 3 Laboratoire TIMC-IMAG, Centre National de la Recherche Scientifique, Université Joseph Fourier, Grenoble, France; 4 Science for Life Laboratory, Uppsala University, Uppsala, Sweden

**Keywords:** Local adaptation, Haplotype, Positive selection, Human height

## Abstract

**Background:**

Genome-wide scans for regions that demonstrate deviating patterns of genetic variation have become common approaches for finding genes targeted by selection. Several genomic patterns have been utilized for this purpose, including deviations in haplotype homozygosity, frequency spectra and genetic differentiation between populations.

**Results:**

We describe a novel approach based on the Maximum Frequency of Private Haplotypes – MFPH – to search for signals of recent population-specific selection. The MFPH statistic is straightforward to compute for phased SNP- and sequence-data. Using both simulated and empirical data, we show that MFPH can be a powerful statistic to detect recent population-specific selection, that it performs at the same level as other commonly used summary statistics (e.g. F_ST_, iHS and XP-EHH), and that MFPH in some cases capture signals of selection that are missed by other statistics. For instance, in the Maasai, MFPH reveals a strong signal of selection in a region where other investigated statistics fail to pick up a clear signal that contains the genes *DOCK3, MAPKAPK3* and *CISH*. This region has been suggested to affect height in many populations based on phenotype-genotype association studies. It has specifically been suggested to be targeted by selection in Pygmy groups, which are on the opposite end of the human height spectrum compared to the Maasai.

**Conclusions:**

From the analysis of both simulated and publicly available empirical data, we show that MFPH represents a summary statistic that can provide further insight concerning population-specific adaptation.

## Background

With the advent of new sequencing and SNP-genotyping technologies, searching for genomic regions affected by selection has become part of a standard population genetic analysis. Various types of selection cause deviations from the neutral expectation in patterns of genetic variation around particular loci under selection (*e.g. *[1]). Several approaches for detecting these regions have been developed, including deviations in haplotype homozygosity, frequency spectra or genetic differentiation between populations. The basic principle often involves computing a summary statistic across the genome and then search for genomic regions that are outliers relative to the genome-wide distribution. Some approaches search for deviations in the allele frequency spectrum [2,3], others focus on extreme patterns of extended haplotype homozygosity [4-6], and some utilize signals of extraordinary population-differentiation (*e.g*. [7]). These methods have varying power to detect signals of selection depending on how far back in time the selection occurred [8].

Many species and populations have been found to have adapted to local environments, such as climate conditions, food resources, and pathogen exposure. Evidence for adaptation to soil conditions have been found in some *Arabidopsis lyrata* populations [9], and adaptation to climate conditions have been found in some *Arabidopsis thaliana* populations [10]*.* Examples of adaptation to local conditions have also been found in animals, including pigmentation variation in mice [11], wing patterns in butterflies, and adaptation to depth in the lake trout [12]. Population-specific selection or local adaptation is typically a recent phenomenon (at least on an evolutionary time-scale), and migration can easily obscure the signal in the genome over time, making signals of local adaptation particularly difficult to detect.

Humans have also been exposed to new environments and living conditions when colonizing new geographical areas and adopting various lifestyles. A handful of regions in the human genomes have been linked to population-specific selection, including lactase persistence connected to the *LCT*-gene region that emerged independently in northwestern Europeans [13] and pastoralist groups in Africa [14,15]; resistance to infections connected to the *CCR5* gene [16]; copy number variation in the amylase gene (*AMY1*) improving the capacity to digest starch-rich diets [17]; genes affecting skin pigmentation in East Asians and Europeans [18]; resistance to malaria [19]; and adaptation to living at high altitudes [20,21]. Studies of local adaptation and the characterization of genome-local patterns of variation among humans may help us to understand the historical and cultural differences among human populations, and may also be informative of different metabolic reactions to medicines and nourishment [22]. Many of these examples of local adaptation have been detected by candidate gene approaches, but with the wealth of genomic data being accumulated, genome-wide scans for selected regions have become feasible.

With strong selection acting on a gene, the favored variant will increase rapidly in frequency in a short enough time so that recombination does not break down the correlation between SNP-variants around the selected variant. This phenomenon tends to decrease genetic diversity around the selected gene – a selective sweep [23] – and create high-frequency haplotypes. If the variant arose (or became frequent starting from a low level) in a particular population, population-specific selection could potentially be detected as private alleles at high frequency. Among the approaches used for detecting selection, only F_ST_, XP-EHH [6] and XP-EHHST [24] explicitly focus on multiple populations to assess local adaptation. In order to capture signals of local adaptation, we developed a new statistic: the Maximum Frequency of Private Haplotypes (MFPH) in subpopulations. MFPH is based on haplotypes, i.e., combinations of SNP-variants along a chromosome for a particular genomic region. We define private haplotypes as haplotypes that are found in the sample from a focal population, which are absent in the samples from other populations. In the analyzes presented in this paper, we require haplotypes to be completely unique to a sample to qualify as private, but this criteria can easily be modified to allow for a low frequency of the same haplotype in other samples (see Material and Methods). We investigate the properties of this statistic using simulations and publicly available data from humans, as well as comparing its performance to other statistics commonly used for detecting selection.

## Results

**Figure 1:**
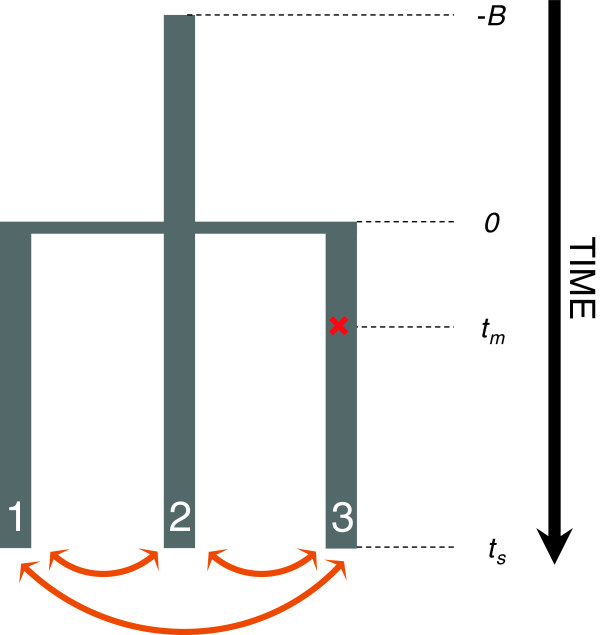
**Scheme of the model used in the simulation study.** An ancestral population of 500 diploid individuals reaches mutation-drift equilibrium during B generations, it then splits into three populations of 500 random-mating diploid individuals each. At time t_m_ a mutation occurs in population 3, which is adaptive in population 3 if G > 0. At time t_s_, 15 individuals are sampled from each of the three populations.

First, we study the behavior of MFPH for simulated data using a population divergence model (Figure 1), both with population specific selection, and without selection (the “neutral cases”). Second, we investigate HapMap III SNP genotype data to validate that MFPH picks up signals at some of the most well-characterized examples of strong population-specific selection in the human genome. Third, we discuss some regions in the HapMap III data that are exclusively picked up by MFPH and not by the other investigated statistics.

### Factors that influence MFPH

To characterize the sensitivity of MFPH to confounding factors, we investigate the impact of various population- and genetic parameters on MFPH. We also compare the performance of MFPH to other statistics used to detect selection.

**Figure 2:**
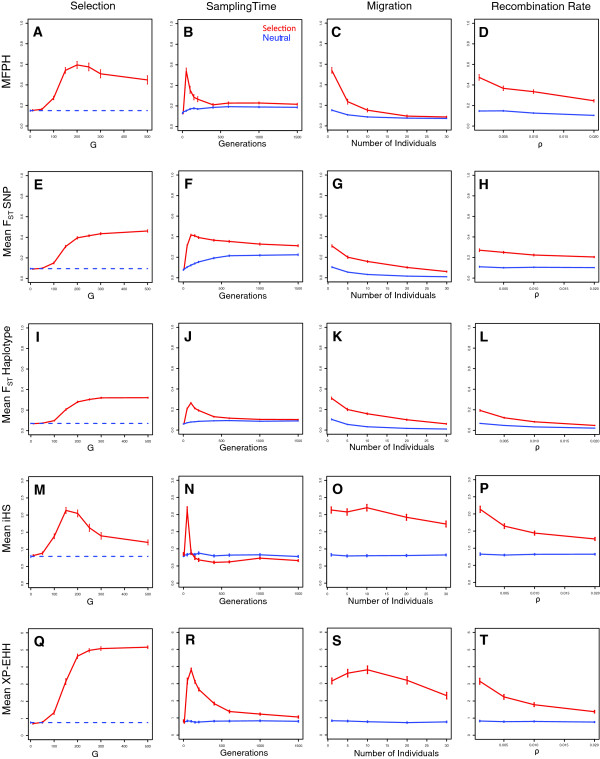
**Effects of various properties on F**_**ST **_**measures, iHS, XP-EHH and MFPH.** Influence of sampling time, migration rate, selection strength and recombination rate in simulations with selection (G > 0) (red line) and simulations without selection (G = 0, blue line). Mean values were calculated on 5 kb-windows containing the variant at site 50,001 and averaged over 100 simulations. Unless variable along the x-axis, the default values for the parameters were: G = 150, ρ = 0.001, m = 1, θ = 0.001, t_m_ = 100, t_s_ = 50, N = 500. **A** to **D**: mean MFPH, **E** to **H**: mean F_ST_ for SNPs, **I** to **L**: mean F_ST_ for haplotypes, **M** to **P**: mean iHS (absolute value), **Q** to **T**: mean XP-EHH.

The strength of selection (*G*) naturally affects the signal of selection and the difference between the selected and neutral cases increases with increasing *G* (Figure 2A). If selection is very strong (G > 250), MFPH starts to decrease, probably due that the selected variant quickly fixed in the focal population and that the beneficial variant spread via migration to neighboring populations (Additional file 1: Table S2 shows that for very high selection coefficients, the most frequent allele in population 3 – the population where selection is acting – is typically almost fixed and not unique to population 3).

The mean of MFPH decreases with sampling time and there is essentially no signal of selection when the sampling occurred more than 200 generations after the emergence of the selected variant (Figure 2B).

Since MFPH is based on private haplotypes, migration will affect MFPH. As shown in Figure 2C, at migration rates above 10 migrants per generation, the difference between cases with and without selection becomes small, and when the migration rate reaches 20, discriminating between the neutral and selected cases becomes difficult (Figure 2C and Additional file 1: Table S2).

**Figure 3:**
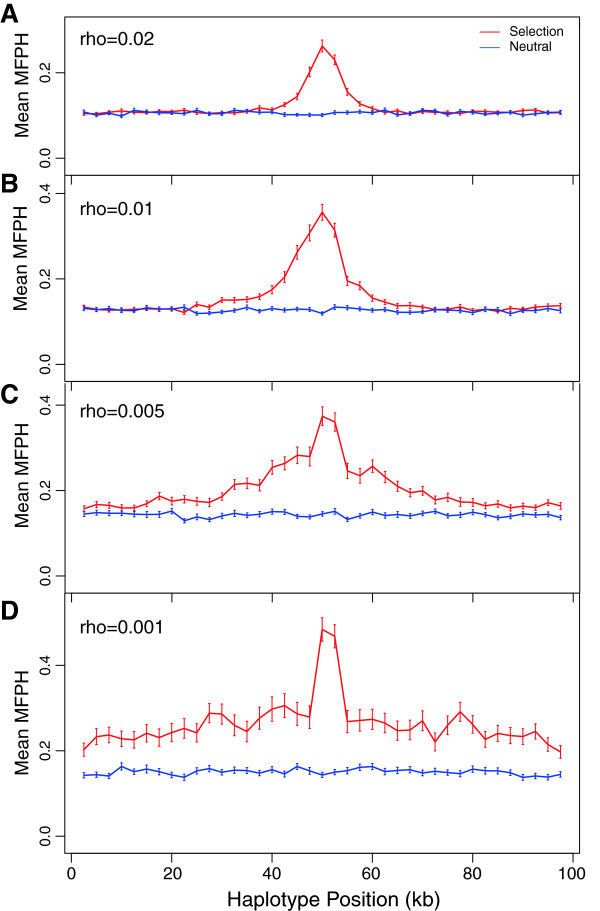
**Effect of recombination rate on MFPH. A** to **D**: MFPH averaged over 100 simulations, for decreasing recombination rate (ρ = 0.02, 0.01; 0.005, 0.001 respectively). The other parameters were: G = 150, m = 1, θ = 0.001, t_m_ = 100, t_s_ = 50, N = 500. Window size: 5 kb, step: 2.5 kb.

Another factor that impacts MFPH is the recombination rate. Simulations with selection, revealed a decrease in MFPH with increasing recombination rate (Figure 2D). However, MFPH was much greater in simulations with selection compared to simulations without selection, even for relatively large recombination rates (Figures 2D and Figure 3). For high recombination rates (low levels of LD, Figure 3A and B), MFPH drops rapidly towards the value under neutrality as the distance from the selected site increases. In contrast, if the recombination rate is low (high levels of LD, Figure 3D), MFPH remains above the level of the neutral case over a much longer region.

**Figure 4:**
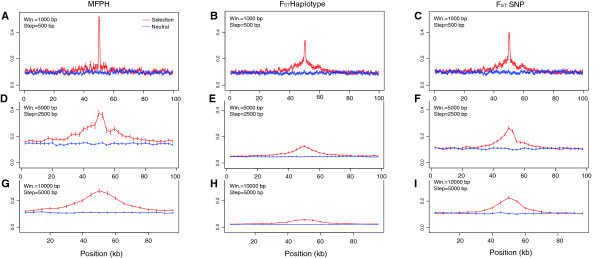
**Effect of the window-size on the three window-size dependent summary statistics.** The figure shows scans along the same data using three different window-sizes: 1, 5, and 10 kb, with step lengths 500 bp, 2.5 kb and 5 kb respectively. Parameters: G = 150, ρ = 0.001, m = 1, θ = 0.001, t_m_ = 100, t_s_ = 50, N = 500. MFPH, Fst SNP and Fst haplotype are averaged over 100 simulations. **A** to **C**: MFPH, **D** to **F**: F_ST_ haplotype, **G** to **I**: F_ST_ SNP.

The choice of window-size also impacts MFPH. For example, as the window-size increases, the magnitude of the peak at the selected site decreases while the width of the peak increases (Figure 4A, D and G). The decrease in MFPH at the selected site is likely an effect of that many distinct low-frequency haplotypes dominate the haplotype-window if the window-size is large and that there is more than one haplotype under positive selection (increasing the recombination rate has a similar effect). This phenomenon is also evident for the F_ST_ measures, in particular F_ST_ based on haplotypes (Figure 4). However, even a ten-fold difference in window-size had a minor impact on the qualitative behavior of MFPH in our simulations (Additional file 1: Figure S3).

### Comparing MFPH to other statistics used for detecting selection

Various summary statistics commonly used to search for signals of selection were also computed based on the same data to compare with MFPH, including iHS [5], XP-EHH [6] and F_ST _[25,26]. We compute two different versions of F_ST_: F_ST_ based on the haplotypes defined by a specific window (which we refer to as “F_ST_ haplotype”) and the average value of F_ST_ across SNPs in a specific window (“F_ST_ SNP”) (see Additional file 1). Other commonly used summary statistics for detecting signals of selection include Tajima’s D [2], and Fay & Wu’s H [3]. These statistics were however only included for completeness since they are not based on haplotypes or specifically designed to detect population-specific selection.

Overall, the factors that influence MFPH have similar effects on F_ST_, iHS and XP-EHH (Figure 2, see Additional file 1: Figure S4 for the behavior of Tajima’s D and Fay & Wu’s H). Sampling time have a relatively small effect on XP-EHH and F_ST_ based on SNPs and the signal of selection can be detected for long time-periods after the emergence of the selected variant (Figure 2). MFPH, iHS and F_ST_ based on haplotypes capture the selection signal well if the selected variant emerged recently (less than 100 generations ago), but fails to detect selection on variants that emerged earlier. Migration has a strong effect on the ability of F_ST_ measures to pick up the selection signal, similar to the behavior of MFPH. In contrast, iHS and XP-EHH can distinguish a selection signal even if the migration rate is substantial. Compared to MFPH and iHS, both F_ST_ measures and XP-EHH are better at distinguishing cases with population-specific selection from neutral cases if the selection coefficient is large. The somewhat poorer performance of MFPH and iHS in this case may be due to the loss in power when the advantageous variant is close to fixation [5,6]. MFPH, iHS and XP-EHH are more sensitive to weak selection (G < 100) while F_ST_ based on haplotypes start to pick up a selection signal only when G reaches 150. All investigated statistics show decreasing power to detect selection with increasing recombination rate. However, even for the greatest recombination rates we investigate here (up to 20 times greater than the mutation rate), the statistics were able to distinguish the cases with selection from the cases without selection (except perhaps for F_ST_ based on haplotypes).

### HapMap III data

We computed MFPH for the following HapMap III populations: Maasai from Kinyawa in Kenya (MKK), CEPH Europeans from Utah of north-western European descent (CEU) and Japanese from Tokyo together with Han Chinese from Beijing (JPT + CHB). These populations were selected to minimize the occurrence of recent migration between populations and because particular population-specific selection events have been described for these populations.

**Figure 5:**
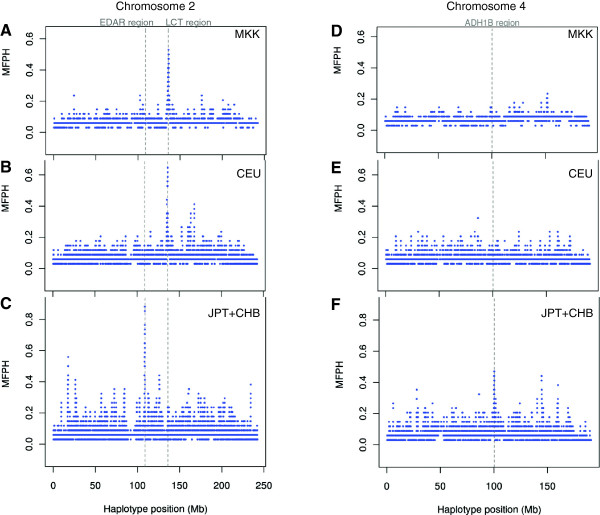
**Genome scan with MFPH of HapMap III phased data.** MFPH is calculated in one population compared to the merged two others. Window-size was set to 200 SNPs with a step length of 1 SNP. **A** to **C**: MFPH across chromosome 2 focusing on MKK, CEU, JPT + CHB respectively, **D** to **F**: MFPH across chromosome 4 focusing on MKK, CEU and JPT + CHB respectively.

Based on these three populations, the greatest genome-wide value of MFPH is located around the *LCT* gene on chromosome 2 in Maasai and north-western Europeans (Figure 5 and Additional file 1: Figure S5-S7), which is consistent with previous results revealing selection for lactase persistence in this region and in these populations [13-15]. Large MFPH values for the East Asian population were found on chromosome 2 and 4, specifically overlapping the *EDAR* gene region on chromosome 2 and the *ADH1B* gene region on chromosome 4, also consistent with previous results [6,27,28]. The distinct MFPH signals around the *LCT* gene region for the Maasai and the north-western Europeans as well as the signal around the *ADH1B* and *EDAR* genes in the East Asian population show that MFPH has power to detect population specific selection events (Figure 5 and Additional file 1: Figure S5-S7, see also Additional fi1e 1: Figures S8-S12 for a comparison of MFPH to XP-EHH, iHS and F_ST_ haplotype in these regions).

The variance of MFPH was greatest for the East Asian population (Additional file 1: Figure S5-S7), followed by the north-western European population and the East African population. This can be a consequence of the demographic history of these populations with well documented bottlenecks affecting the Asian and the European populations [29-31]. The choice of reference populations and sample size also affects MFPH. For instance, computing MFPH across the genome for all ten HapMap III populations results in that the MFPH-signal disappears around the LCT-gene region in the north-western European population (Additional file 1: Figure S13). This effect of pooling is not surprising considering that several of these populations have similar genetic background, and haplotypes are likely to be shared across these populations (e.g. between the north-western European population (CEU) and the British population (GRB)), which will impact statistics that rely on population differentiation (like MFPH, XP-EHH and F_ST_). This also illustrates that conducting scans for local adaptation on different sets of populations can, in fact, provide information about the nature of the selective event.

Finally we investigated the top MFPH signals after excluding chromosome 2 (on which both *EDAR* and *LCT* are located) in the three populations. For the European sample, eleven windows were in the extreme top tail (8.86*10^-6^ tail) and had an MFPH value of 16/34 (1 window) and 15/34 (10 windows) (the exact ratios are due to that MFPH has a discrete set of possible values with *n* + 1 possible values for a sample of size *n*). For the African sample, 75 windows (corresponding to the 6.04*10^-5^ tail) had values of 11/34 (1 window) and 10/34 (74 windows) and 42 windows (corresponding to the 3.38*10^-5^ tail) had a value of 25/34 for the Asian sample. These candidate windows were often adjacent to each other in each population and clustered into two regions for the European and the African sample and one region for the Asian sample (Table 1, see also Additional file 1: Figure S5). As we were specifically searching for windows where MFPH showed a strong signal while there was little signal in the other investigated statistics we focused on the two African Maasai candidate windows on chromosome 3 for which there was little evidence of selection based on iHS, XP-EHH and the two F_ST_ measures. One of these regions is located on chromosome 3 between 50.6 and 51.3 Mb and contains, *inter alia,* the genes *CISH* (cytokin induced STAT inhibitor), *MAPKAPK3* (MAP kinase-activated protein kinase 3, Ser/Thr kinase) and *DOCK3* (dedicator of cytokinesis 3) – all potentially affecting height [32] (Additional file 1: Figure S14A). The other candidate region is between 101 and 101.4 Mb (on chromosome 3) containing the genes *IMPG2* (interphotoreceptor matrix proteoglycan-2), *SENP7* (SUMO1/sentrin specific peptidase 7) and *PCNP* (PEST proteolytic signal containing nuclear protein; Additional file 1: Figure S14B).

**Table 1:** The regions with the highest MFPH value across the Hapmap III data after excluding chromosome 2

**Population**	**Region**	**Genes**
CEU	Chr10:74,416,452-75,102,866	MCU, OIT3, PLA2G12B, NUDT13, ECD, DNAJC9, MRPS16, TTC18
CEU	Chr6:145,190,620-145,554,235	
MKK	Chr3:100,979,041-101,375,515	IMPG2, SENP7, PCNP
MKK	Chr3:50,617,979-51,354,540	HEMK1, CISH, MAPKAPK3, DOCK3
JPT + CHB	Chr15:62,232,223-62,888,060	TLN2, VPS13C, C2CD4A, C2CD4B

In the CEU population, a region located around 74 Mb on chromosome 10 show a peak in MFPH (Additional file 1: Figure S5 and S15). This region contains the genes (among others) *MCU* (mitochondrial calcium uniporter), MRPS16 (human mitochondrial ribosomal protein S16) and *PLA2G12B* (shown to be important for HDL cholesterol levels in mice [33]).

## Discussion

In this study we present a new haplotype-based statistic for detecting population specific positive-selection, which is intuitive and easy to compute. We compare the behavior of MFPH to similar and commonly used summary statistics for detecting selection, including F_ST _[25], XP-EHH ([6], see also [24] for an additional example of a similar statistic) and iHS [5]. These summary statistics have often been used in scans for regions targeted by selection relying on an outlier approach. The conceptual idea of the outlier approach is that if there are regions targeted by selection – but that these are relatively rare– these regions are likely to show up as outliers compared to the genome-wide distribution. These outlier-regions are therefore potential targets for selection, although it is difficult to assess significance for a set of identified outliers to be true targets for selection (see *e.g. *[34-36]).

**Figure 6:**
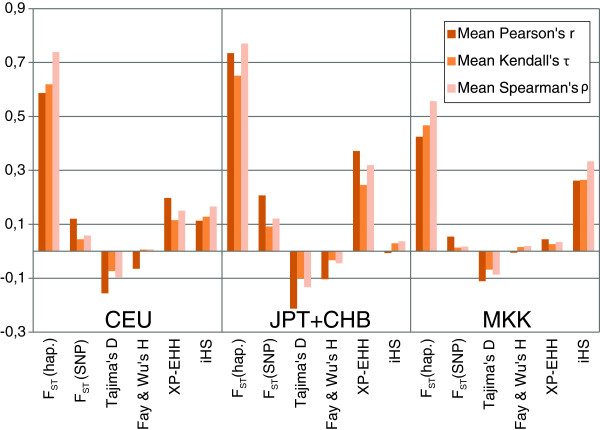
Correlations between MFPH and the window mean of different summary statistics for HapMap III phased data using a window-size of 300 SNPs with a step length of 1 SNP.

Using both simulations and empirical data we conclude that MFPH has similar power for detecting selection compared to many other summary statistics (Figure 2 and Additional file 1: Figure S4). We show that MFPH detects a clear signal of selection in some of the most well-known examples of selection in the human genome: the *LCT* gene-region in Maasai and north-western Europeans and *EDAR* and *ADH1B* in East Asians (Figure 5). Using genome-wide correlations we find that MFPH correlates the strongest with haplotype based F_ST_ followed by either XP-EHH or iHS depending on the population considered (Figure 6). This population dependency illustrates that MFPH is an additional source of information compared to iHS, XP-EHH and F_ST_.

An MFPH scan of the Hapmap III data revealed five top regions, two in the Maasai, two in the European sample and one region in the Asian sample (Table 1). The two regions in the Maasai (both on chromosome 3) were not captured by any of the other statistics and one of these regions, the region around position 51 Mb, (Additional file 1: Figure S14A) coincides with a region that has been implicated as a target for selection on stature in Pygmy groups [32]. While the average stature within Pygmy populations is exceptionally short compared to other African populations [37], the Maasai are among the tallest [38]. Interestingly the Pygmy populations and Maasai show distinctly different genotypes in this region (Additional file 1: Figure S16) suggesting that different haplotypes in the region have been targeted by selection for stature in the Maasai and the Pygmy populations. There are three genes associated with variation in height in this region [32]: *DOCK3*, a guanine nucleotide exchange factor that has been associated with height variation in Europeans [39], the *CISH* gene which has been shown to inhibit growth factors [40] and *MAPKAPK3,* involved in growth, development and stress [41]. This region has a low level of LD (and hence a small genetic distance (in cM) for LD-based genetic maps such as the HapMap genetic maps). Indeed, if windows based on cM are used to compute MFPH, this particular region would not be a top candidate in MKK (see Additional file 1: Figure S7). However, since one of the most characteristic signals of selective sweeps is high LD, using windows from LD-based recombination maps will likely result in substantial loss of power for any haplotype based statistic targeting selective sweeps. Indeed, the *LCT* region also has high LD (and small genetic distances in cM for HapMap recombiantion maps) due to recurrent selective sweeps (at least two selective sweeps occurred in the LCT region [13-15]). For MFPH one can choose to control for diversity (using SNP-windows) or control for recombination rate (using cM-windows; see Additional file 1 for correlations between MFPH and genetic distances).

The region situated around 101 Mb on chromosome 3 (Additional file 1: Figure S14B) includes the gene *IMPG2* which codes for an interphotoreceptor matrix proteoglycan. This gene has been pointed out as important in diabetic retinopathy [42,43] and thus possibly also involved in other types of retinopathies such as solar retinopathy. Although little is known about the molecular mechanisms of solar retinopathies, individuals with greater exposure to sunlight show greater frequency of solar retinopathies [44] which could potentially have led to adaptation targeting the *IMPG2* gene among the Maasai as an effect of exposure to sunlight and UV radiation (at least compared to the comparative European and Asian populations). While the 51 Mb-region (chromosome 3) has been implicated as a target for selection before, neither of these regions on chromosome 3 would have been found and highlighted as candidate regions for local adaptation in the Maasai based on iHS, XP-EHH or the two F_ST_ measures.

Similarly, in CEU, the MFPH peak around 74 Mb on chromosome 10 (Additional file 1: Figure S15) may indicate that this region has been under recent positive selection while there is little indication of this based on the other statistics. This region contains at least two interesting genes, *P4HA* and *PLA2G12B. PLA2G12B* codes for a phospholipase initially shown to be lacking activity [45] but also to be involved in HDL cholesterol level in mouse [33]. *PLA2G12B* is a member of the PLA2 group of genes that are globally involved in many mechanisms like lipid digestion, inflammation and degradation of bacterial phospholipids (cited in [46]). *P4HA* is responsible for the synthesis of collagen and is interestingly expressed in macrophages and thus probably involved in the repair of injured or inflamed tissues [47]. Thus, though more information is required, there is some evidence that alleles of these two genes could have for been targets of selection among Europeans in response to pathogen exposure.

**Figure 7:**
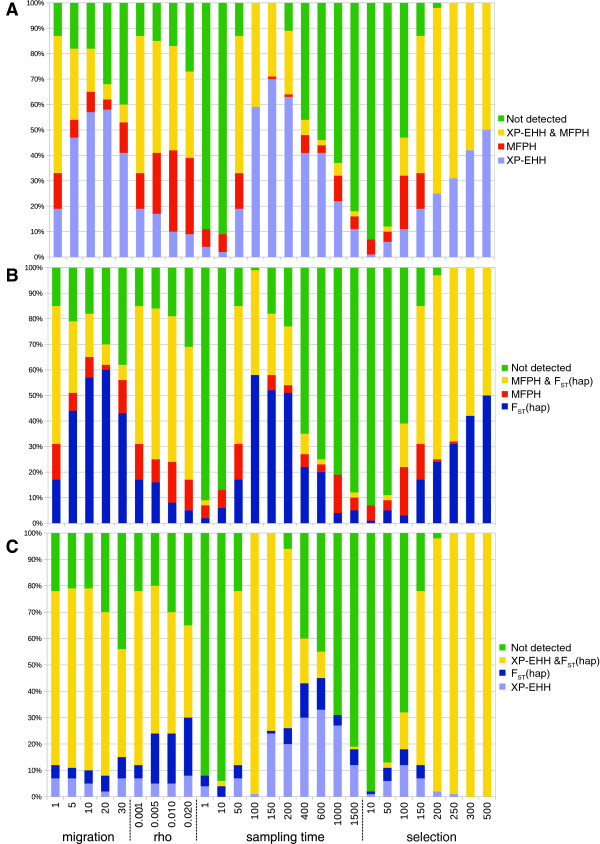
**Overlap of summary statistics when they are two standard deviations away from the mean in the simulated data.** Default parameter values were G = 150, ρ = 0.001, m = 1, θ = 0.001, t_m_ = 100, t_s_ = 50, N = 500. Mean and standard deviation were calculated on the corresponding neutral simulations (same parameters but with G = 0). **A**: MFPH and XP-EHH, **B**: MFPH and F_ST_ haplotype, **C**: XP-EHH and F_ST_ haplotype.

To closer assess the additional information contained in MFPH relative to XP-EHH and haplotype based F_ST_ in the presence of selection, we used simulations with population-specific selection. We used two standard deviations from the hypothetical genome-wide mean (here represented by simulations where the selection coefficient is set to zero) for each summary statistic as the indicator of selection. This set-up allowed us to quantify how often MFPH detects (or fails to detect) a signal of selection that was detected (or not detected) by XP-EHH or F_ST_ Haplotype (Figure 7). There were many cases when MFPH finds a (true) signal of selection which was missed by the alternative statistics (XP-EHH or F_ST_) implying that MFPH provides additional information, and there were also many cases when either XP-EHH or F_ST_ detected selection while it was missed by the other statistics (Figure 7). In the simulations with very strong selection, MFPH detected a subset of cases compared to either XP-EHH or F_ST_. Interestingly, there seemed to be considerably less overlap in signal between MFPH and either F_ST_ or XP-EHH than between XP-EHH and F_ST_ suggesting that combining either XP-EHH or F_ST_ with MFPH may capture a larger set of the selection-cases compared to the combination of XP-EHH and F_ST_ (Figure 7).

MFPH depends on the choice of populations being contrasted. Since it is based on population-specificity, comparing recently diverged populations or admixed population will decrease the power of MFPH, but it is easy to adjust the computation of MFPH to allow some level of haplotype sharing among populations. Contrasting a focal subpopulation to a few selected populations in the HapMap III or to all HapMap III populations resulted in different outcomes. While some signals remained regardless of the choice of populations, other signals were lost if a larger set of populations were used (Additional file 1: Figure S13), which can be understood by considering the relationship of the populations. This type of information can also be used to investigate (for instance) the age of the selective event as well as pinpointing which particular populations have been affected by selection.

MFPH also depends on the choice of window-size (Figure 4). In theory, the strength of selection, the time since the selection started and the recombination rate should govern the expected width of the region around a selected site that retains a signal of deviation from the genome-wide average. In other words, the size of a deviant region should contain information about the nature of the selection event. For example, since MFPH is straight-forward to compute for various choices of window-sizes, the effect of window-size can be integrated into the statistical framework (somewhat similar to the wavelet-transform analyzes in [48]) and help determine properties of detected selection signals.

The rapidly increasing amount of sequence data will be ideal to investigate using MFPH. For example, variants at low frequency (e.g. caused by sequencing errors or rare variants) will typically not influence the most frequent haplotype and therefore not MFPH either. For the same reason is MFPH not likely to be efficient at detecting background selection or negative selection. MFPH is further only marginally affected by phasing errors (Additional file 1) as phasing errors typically create low frequency haplotypes [49]. Compared to sequence based statistics such as Tajima’s D and Fay & Wu's H, MFPH also shares the feature with other haplotype based statistics of being less affected by SNP ascertainment biases [50] making it an ideal statistic for SNP data or low-coverage sequence data that fails to capture all variants. Finally, differences in variance of MFPH across populations suggest that demographic events influence MFPH to some degree and the effect of demography on MFPH should be assessed for investigations of specific populations (e.g. [51]).

## Conclusions

Our simulation studies of population specific selection under various model parameters as well as comparisons to other summary statistics show that MFPH is a powerful tool for detecting recent, relatively strong population-specific selection. We demonstrate that MFPH has similar power to F_ST_ and XP-EHH (two similar and widely used statistics). Importantly, MFPH may capture events that are missed by other statistics. For instance, MFPH alone implicated selection in a gene-region in Maasai that has been pointed out as a candidate region for stature in Pygmy groups. Thus, MFPH constitutes a valuable additional summary statistic for investigating local adaptation, possibly in a demography-informed approach utilizing, for instance, Approximate Bayesian Computation [52,53]. MFPH is well suited for analyzing large genome wide data since it is quick and easy to compute for phased data. Moreover, since MFPH is defined in terms of haplotypes, it is expected to be robust to effects of ascertainment bias and because it focuses on the maximum frequency of haplotypes, it should also be robust to phasing and sequencing errors that create rare haplotypes.

## Methods

### Definition of MFPH

We focus on haplotypes, i.e., combinations of SNP-variants along a chromosome for a particular genome region. We define private haplotypes as haplotypes that are found in the sample from one particular population, but absent in the samples from other populations. Note that “private” is sample based and that a private haplotype can potentially be present in more than one population. Sample size affects the probability of sampling alleles, and in the case of unequal sample sizes, the rarefaction approach can be used to obtain comparable statistics [54,55], or, alternatively, down-sampling can be employed to obtain comparable sample sizes.

Formally, let *n*_*i*_ denote the number of sampled sequences from population *i* (*i = 1 … S*). Focus on a locus *l* in a sequence (a predefined window of either a specific number of consecutive SNPs or a specified length of a region in either base pairs or centimorgans). Let *h(i,j,l)* denote the haplotype of sequence *j* in the sample from subpopulation *i* at locus *l*. A haplotype *x* is defined as *private* to population *k* at locus *l* iff:

0 ≤ sample frequency of x after excluding the sample from subpopulation k ≤ ϵ < sample frequency of x in subpopulation k

or

where I is an indicator variable so that I(True) = 1 and I(False) = 0. Setting *ϵ* = 0 implies that a haplotype is private to population *k* if and only if it is absent in all samples except the sample from population *k* while letting *ϵ* > 0 allows for a less strict definition of privacy. Let *H(k,l)* denote the set of haplotypes private to population *k* at locus *l*, then

$$\mathrm{MFPH}\left(k,l\right)=\max_{x\in H\left(k,l\right)}\frac{\overset{n_{k}}{\underset{j=1}{\Sigma }}I\left(h\left(k,j,l\right)=x\right)}{n_{k}}$$

If *H(k,l)* is empty *MFPH(k,l)* is defined to be 0.

### Model description and simulations

In order to investigate the behavior of MFPH, we simulated genomic data where a specific locus is under positive selection using forward simulations implemented in the software SFS_code [56]. We model three populations of equal size (*N* = 500, diploid individuals) that split from an ancestral population (*N* = 500, diploid individuals) at time zero. The population size has been chosen arbitrarily in order to have reasonable computation times. Each simulated individual is represented by two chromosomes of length (*L*) 100,000 bp. Individuals can migrate between populations at rate *m* which represents the number of individuals coming from the two other populations into one particular population each generation. Sites mutate with a population-scaled per site rate of *θ* (*θ =4Nμ*, where *μ* is the per-site per generation mutation rate). Mutations occur under a pseudo-infinite site model (see the SFS_code documentation [56] for more details). Recombination events occur with a population-scaled rate of *ρ* (*ρ = 4Nr*), where *r* is the probability of cross-over between two adjacent sites per generation. The population size scaled mutation rate per site was set to *θ* = 0.001 (implying a scaled mutation rate for the fragment θ_L_ = 100) and the recombination rate *ρ* was set to values between 0.001 and 0.02 (the scaled recombination rate for the fragment, *ρ*_*L*_, was set between 100 and 2000). Assuming a mutation rate of 1.25 × 10^-8^ per site per generation [57], our simulated θ_L_ = 100 corresponds to a 4 Mb DNA fragment in a population of 500 (4 × 500 × 1.25 × 10^-8^ × 4 × 10^6^ = 100) or, alternatively, a 200 kb DNA fragment in a population of 10,000 (4 × 10^4^ × 1.25 × 10^-8^ × 2 × 10^5^ = 100). Since we are only interested in the variable sites, this simplification allowed faster simulations while producing realistic SNP and haplotype data. See Additional file 1: Table S1 for parameter settings of the model.

In order to be able to compare MFPH across independent simulations of the same model, we computed MFPH on bp-windows on simulated data.

In the simulations, a mutation occurs in population 3 at the center of the chromosome (position 50,001 bp) at a fixed time *t*_*m*_ – the “mutation time”, given in number of generations after the population split. Individuals in population 3 carrying the derived variant at this site have a selective advantage with a population-scaled selection coefficient *G.* Individuals in population 1 and 2 carrying this variant do not confer a selective advantage. Samples are drawn after an additional *t*_*s*_ generation following *t*_*m*_ (*t*_*m*_ + *t*_*s*_ generations after the population split). We refer to *t*_*s*_ as the “sampling time”, see Figure 1 for an outline of the model. The ancestral population is allowed to evolve for 5,000 generations (this is a “burn-in time” to omit any effects of the starting conditions, see SFS_code manual) prior to the population split. Conditional on that site 50,001 is polymorphic in the pool of the three populations at the time when the samples are drawn, we generated 100 simulations for each set of model parameters, and averaged the results across simulations. For each set of parameter values we also performed 100 comparative simulations without selection (G = 0) (the “neutral” cases), where we still conditioned on that site 50,001 was polymorphic in order to generate simulated data where the neutral and selected cases were as similar as possible. This conditioning likely had a minor influence on our results: the frequency of the deterministic mutation in population 1 and 2 when it was under selection in population 3 (G > 0) was similar to the frequency in population 3 when there was no selection (G = 0, Additional file 1: Figure S1). In contrast, the frequency of the selected variant in population 3 was markedly increased when G > 0 (Additional file 1: Figure S1). However, to further investigate whether this conditioning had a large influence on the neutral distribution, we performed 10,000 neutral simulations without this conditioning with the (relevant) default parameter values. We compared the distribution of MFPH in a window overlapping the position of the inserted mutation when this mutation was present to when it was absent (Additional file 1: Figure S2). The distributions are similar and we conclude that conditioning on a mutation in the neutral simulations has little or no influence on MFPH.

### Computing MFPH for the HapMap III data

We computed MFPH for the HapMap III phased data [58]. MFPH was calculated for sets of three populations after down-sampling the number of chromosomes to equal the sample size of the population with the smallest sample size. We computed MFPH (and the comparative statistics) for windows with a fixed number of SNPs, a fixed physical-size, and with a fixed size of windows in cM based on the HapMap II genetic map [59] (calculated on the combined CEU, YRI and JPT + CHB populations) with a step-size of one SNP between windows.

To study the effect of how windows are defined, we computed the pairwise correlations between MFPH with a fixed number of base pairs (bp-windows) and MFPH with windows of a fixed genetic distance (cM-windows). For ease of comparison, sizes of bp-windows and cM-windows were chosen according to the mean base pair-size and cM-size of a 200 SNP-window on chromosome 2. Spearman and Pearson correlations were computed between MFPH based on SNP-windows, bp-windows and cM-windows and we found that SNP based windows, bp-windows, cM-windows are highly correlated (between 0.60 and 0.88 depending on the comparison). All three types of windows have respective advantages and disadvantages, and we present the results for windows with a fixed number of SNPs in the main text and based on bp-windows and cM-windows in the supplementary material.

## Competing interests

The authors declare no competing interests.

## Authors’ contributions

MJ and AS conceived the study, AS, PS and MJ development the methods, AS and PS performed the simulations and analyzed the data. AS, PS, and MJ interpreted the results and wrote the paper. All authors read and approved the final manuscript.

## Supplementary Material

Additional file 1 Contains supplementary methods, results, figures and tables. Click here for file
