# Supplementary material for: Private haplotypes can reveal local adaptation
Source: BMC Genet. 2014 May 22;15:61. doi: 10.1186/1471-2156-15-61 (PMC4040116; doi:10.1186/1471-2156-15-61)
Supplement: Additional file 1 — Contains supplementary methods, results, figures and tables. [file 1471-2156-15-61-S1.pdf]

Supplementary Material

Details on computation of F_{ST}

In the materials and methods, we refer to two types of F_{ST}-measures based on the same equation (5.3, p174 in Weir (1996)). "F_{ST}-SNP" uses the equation considering bi-allelic loci in one window, while "F_{ST}-haplotypes" uses the equation considering a window as a haplotype-locus and the haplotype variants as different alleles of this locus. Following Weir (1996), we have

$$\hat{F}_{ST} = \frac{\sum_{l=1}^{m}\sum_{u=1}^{v} T_{1lu}}{\sum_{l=1}^{m}\sum_{u=1}^{v} T_{2lu}}$$

where T_1 and T_2 are described in Weir (1996). In the case of "F_{ST}-SNP", m=number of SNPs in the window and v=2, while in "F_{ST}-haplotypes", m=1 (one window) and v=number of distinct haplotype alleles observed in the window.

Effect of Phasing on MFPH

Methods:

We mixed the known phase of the simulated data according to a binomial random sampling of the SNP alleles of each individual. We phased the data using fastPHASE (Scheet and Stephens 2006) and with the following command:

fastPHASE_Linux -T10 -u(labels.txt) -o(output) file.inp

The phasing was performed for each subpopulation separately and for simulated data from both with and without selection for different values of rho. We counted the cases where the window containing the inserted mutation (under selection) had an MFPH value greater than the mean of the neutral cases plus 2 standard deviations.

Results:

In the simulations without selection (the neutral case), we observe around 5% of false positives regardless of using the known phase or for the statistically phased data (Table S3). For the selection case, we see a substantial overlap of significant MFPH values between the rephased data and in the known-phase data. We note that there are a few more significant cases in the rephrased data compared to the known-phase data. This may be due to phasing the data within sub-populations. We also note that increasing the recombination rate has basically no effect on the difference of the number of significant cases (between known-phase and statistically phased data).

Investigating the effect of differences in recombination maps between populations

Methods

We used the recombination maps available from 1000 Genomes (based on the 2.5M SNP data in CEU and MKK). The CEU and the MKK maps show very similar patterns of recombination. However, to investigate whether a difference in recombination rate between the focal population and the reference population would lead to inflated MFPH values, we calculated the correlation between the MFPH (computed for 415 kbp windows) and the differences in genetic distance (in cM) between the CEU and the MKK (for the same 415 kbp windows). An issue when trying to disentangle such an effect from the already noted negative correlation between recombination rate and MFPH is if a larger difference in recombination rate between the two populations is expected in high-recombination regions. If this is the case, we should scale the difference by dividing by the mean recombination rate in the two populations while, on the other hand, if this is not the case one should study the difference. Hence, for each bp-window we calculated the genetic distance in cM based on the genetic map from i) the focal population and ii) the reference population. Referring to the former by d_f and the latter by d_r, we calculated the correlation of MFPH to both d_f-d_r and to $(d_f$-$d_r)/(0.5(d_f+d_r))$. This computation was performed with CEU as the focal population as well as with MKK as the focal population. The results are shown in Tables S4 and S5. The choice of d_f-d_r or $(d_f$-$d_r)/(0.5(d_f+d_r))$ has little effect and

the correlation between MFPH and difference in genetic distance is small (both using CEU and MKK as focal population).

Supplementary Figures & Tables:

Figure S1: Allele trajectories for the deterministic mutation at site 50,001 in population 3. The parameter settings were ρ=0.001, m=1, θ=0.001, t_m=100. N=500. **A:** Simulations without selection (G=0). **B:** Simulations with selection (G=150). Note that there is no selection for the deterministic mutation in population 1 and 2.

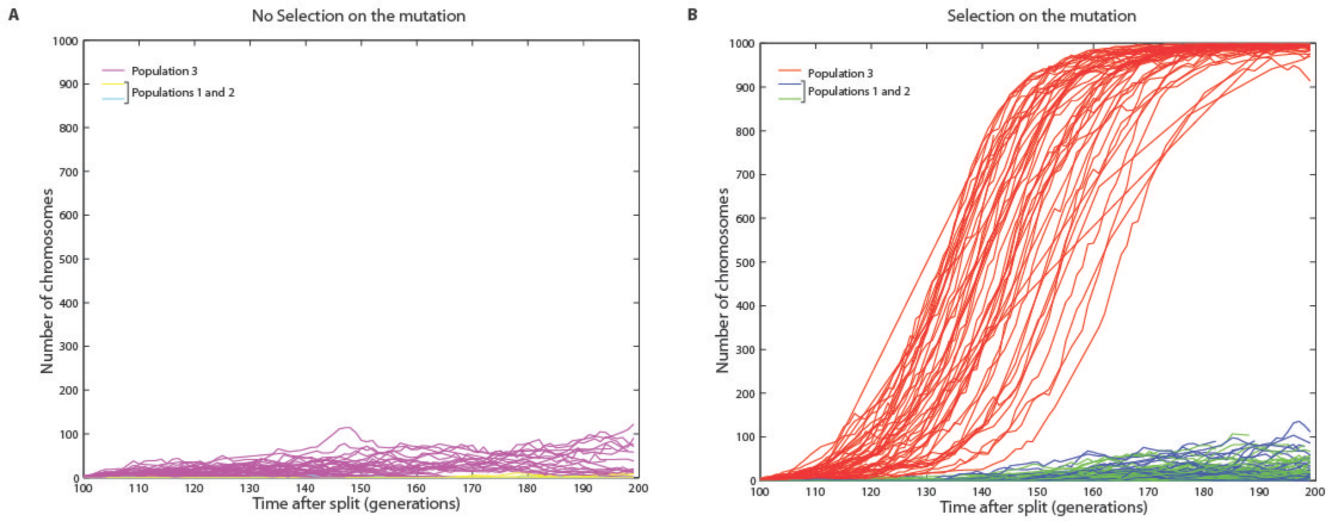

Figure S2: Distribution of MFPH values in neutral simulations. "no mutation": MFPH values for 10,000 simulations in population 3 with no mutation occurring at t_m, "population 3": MFPH values for 100 simulations in population 3 conditioning on a mutation occurring at t_m, "population 1": MFPH values in population 1 for the same simulations as for "population 3". The window considered corresponds to 5kb from position 47.5kb, the mutation position is 50,001bp. ρ=0.001, m=1, θ=0.001, t_m=100, t_s=50, N=500 and G=0.

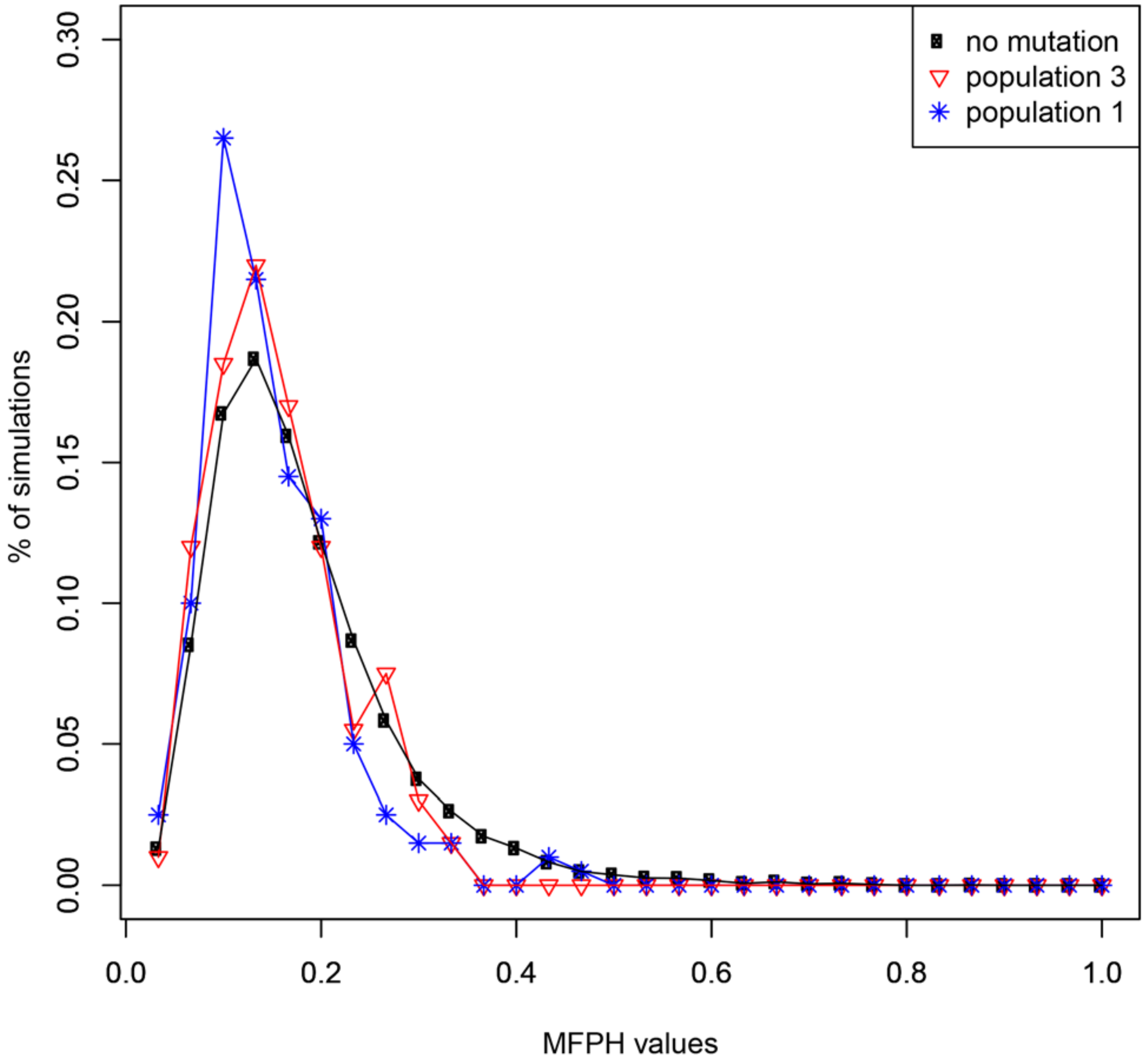

Figure S3: Influence of parameters on MFPH calculated with 1kb and 10kb window-sizes with default parameters G=150 (G=0 for neutral simulations), ρ=0.001, m=1, θ=0.001, t_s=50, t_m=100, N=500. Mean MFPH over 100 simulations was calculated on a window containing site 50,001. **A to D:** window-size = 1 kb. **E to H:** window-size =10 kb.

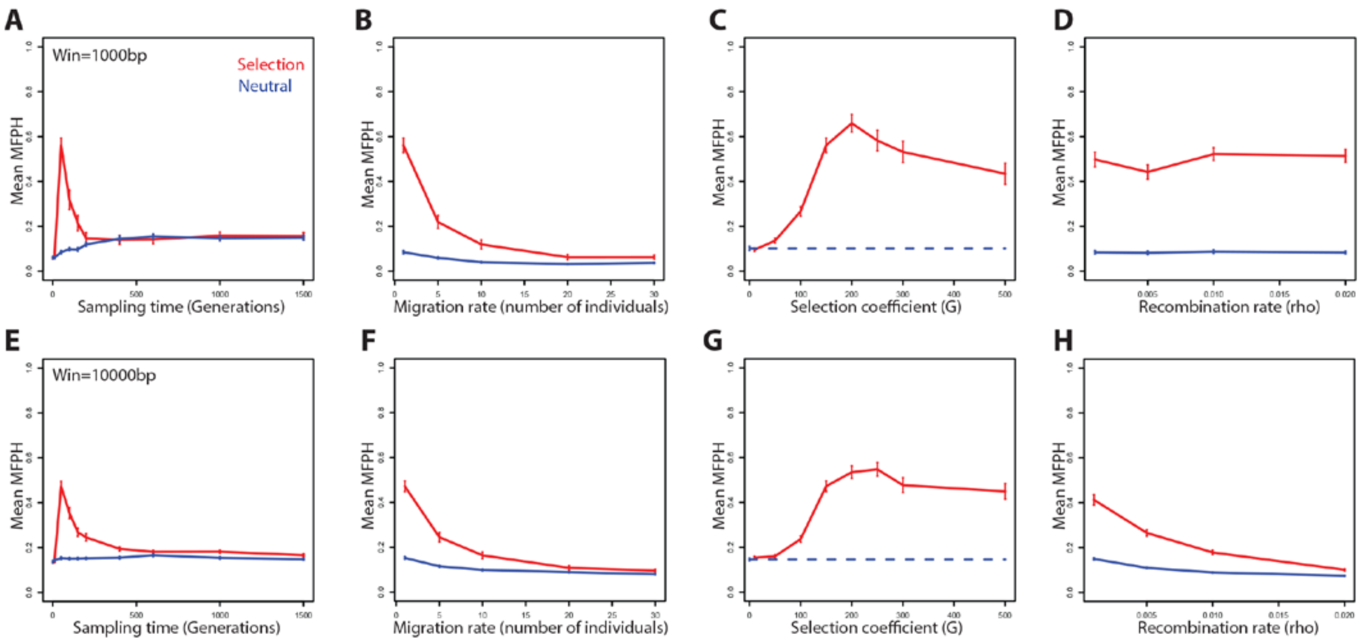

Figure S4: Influence of parameters on Tajima's D and Fay and Wu'H computed on simulated data. Mean values over 100 simulations were computed for a 5 kb window surrounding site 50,001 with default parameters G=150 (G=0 for neutral simulations), ρ=0.001, m=1, θ=0.001, t_s=50, t_m=100, N=500. Red lines represent simulations with selection and blue lines simulation without selection. **A to D:** Fay & Wu's, **E to H:** Tajima's D.

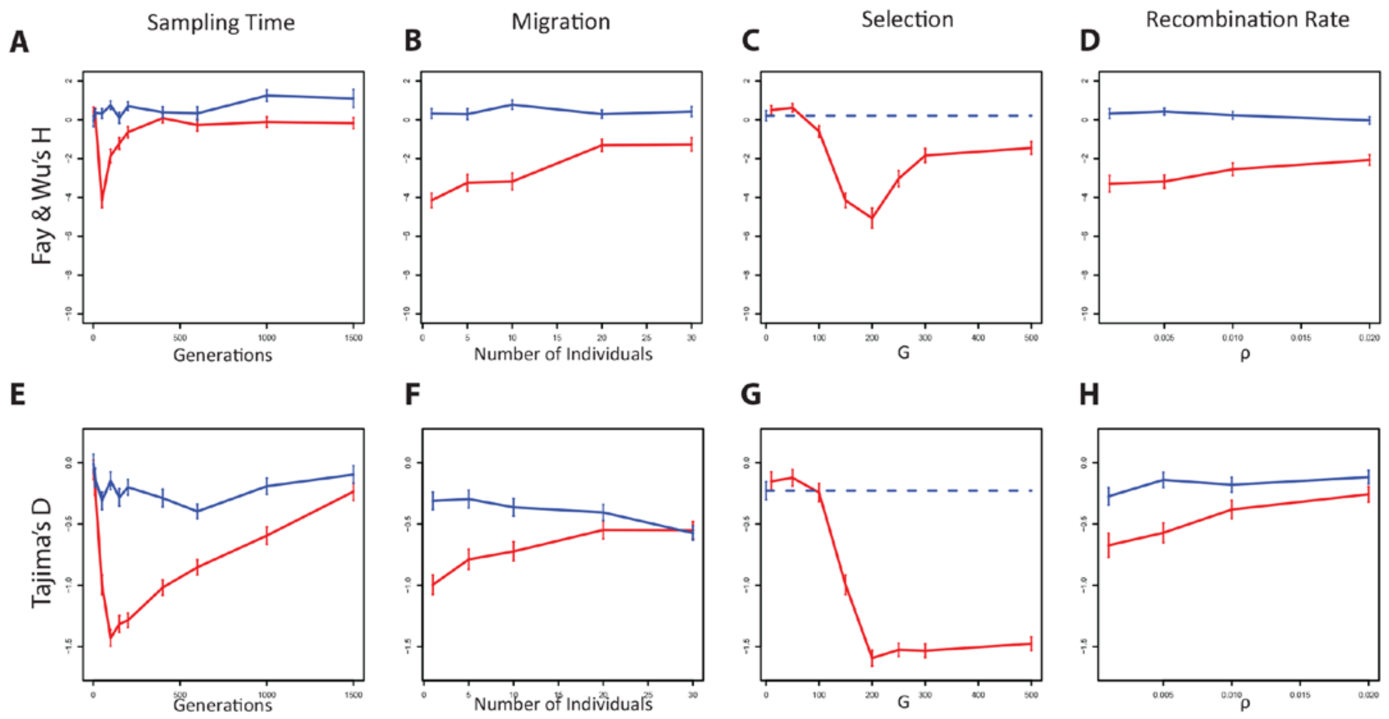

Figure S5: MFPH on HapMap III phased data for chromosome 1-22. Odd numbered chromosomes are represented by light blue and even numbered chromosomes by dark blue. The size of the window was set to 200 SNPs with a 1 SNP step length between windows. The genomic positions of the genes LCT, EDAR (both on chromosome 2) and ADH1B (on chromosome 4) are indicated. The position with a star represents the region of DOCK3-gene. MFPH is calculated in one population (with sample size 34 chromosomes or 17 individuals) compared to the merged two others (34 individuals).

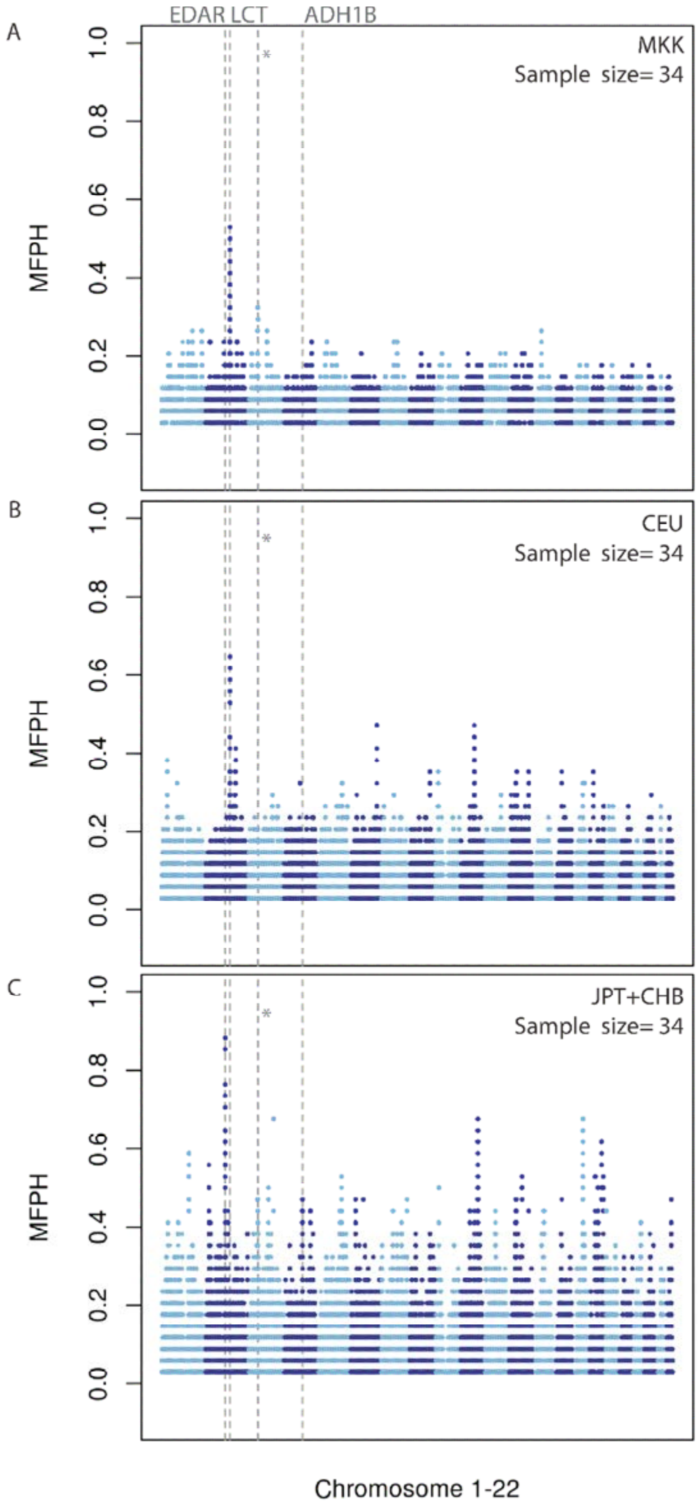

Figure S6: MFPH on HapMap III phased data for chromosome 1-22. Odd numbered chromosomes are represented by light blue and even numbered chromosomes by dark blue. The size of the window was set to 415 kbp with a 1 SNP step length between windows. The genomic positions of the genes LCT, EDAR (both on chromosome 2) and ADH1B (on chromosome 4) are indicated. The position with a star represents the region of DOCK3-gene. MFPH is calculated in one population (with sample size 34 chromosomes or 17 individuals) compared to the merged two others (34 individuals).

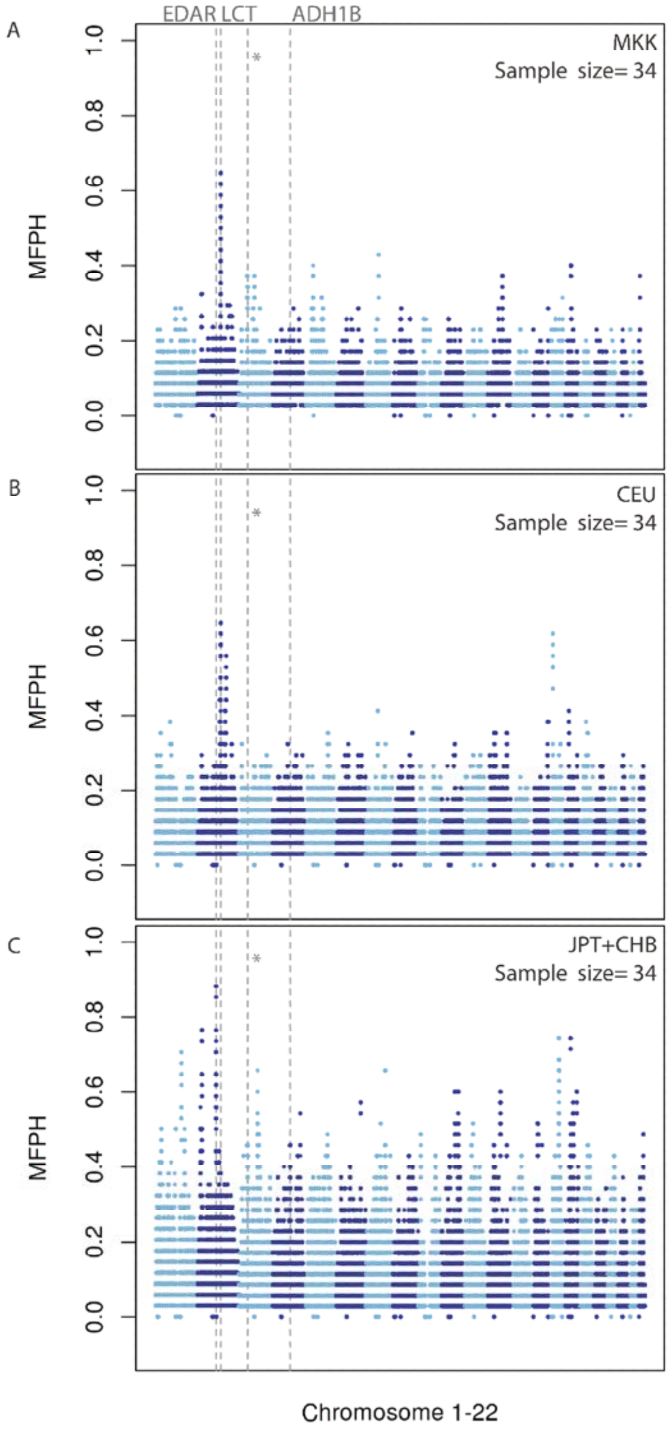

Figure S7: MFPH on HapMap III phased data for chromosome 1-22. Odd numbered chromosomes are represented by light blue and even numbered chromosomes by dark blue. The size of the window was set to 0.452 cM with a 1 SNP step length between windows. The genomic positions of the genes LCT, EDAR (both on chromosome 2) and ADH1B (on chromosome 4) are indicated. The position with a star represents the region of DOCK3-gene. MFPH is calculated in one population (with sample size 34 chromosomes or 17 individuals) compared to the merged two others (34 individuals).

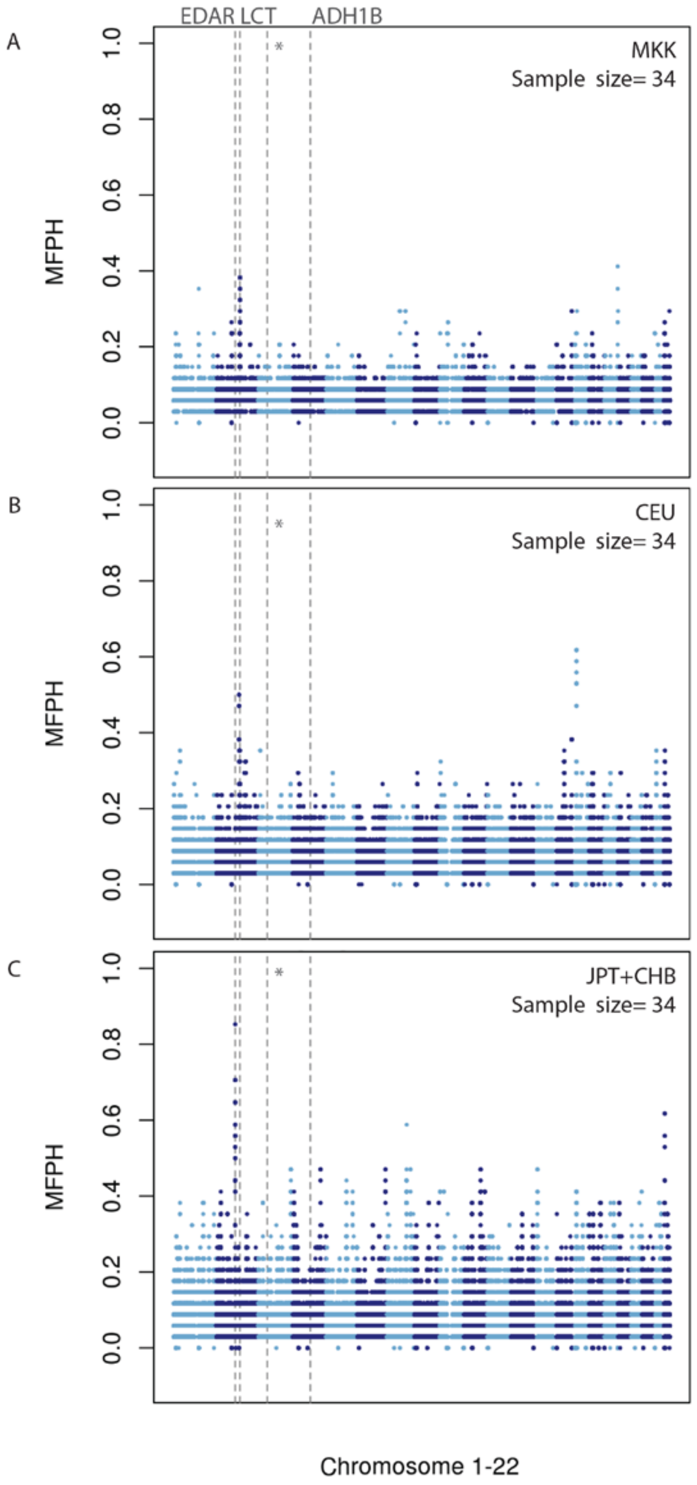

Figure S8: F_{ST} based on the three populations CEU, JPT+CHB, MKK (all down sampled to 17 individuals) for HapMap III phased data. Window-size=200 SNPs, step length=1 SNP. The genomic positions of the genes LCT, EDAR (both on chromosome 2) and ADH1B (on chromosome 4) are indicated. **A and B:** chromosome 2**, C and D:** chromosome 4**. A and C:** F_{ST} Haplotypes, **B and D:** F_{ST} SNPs.

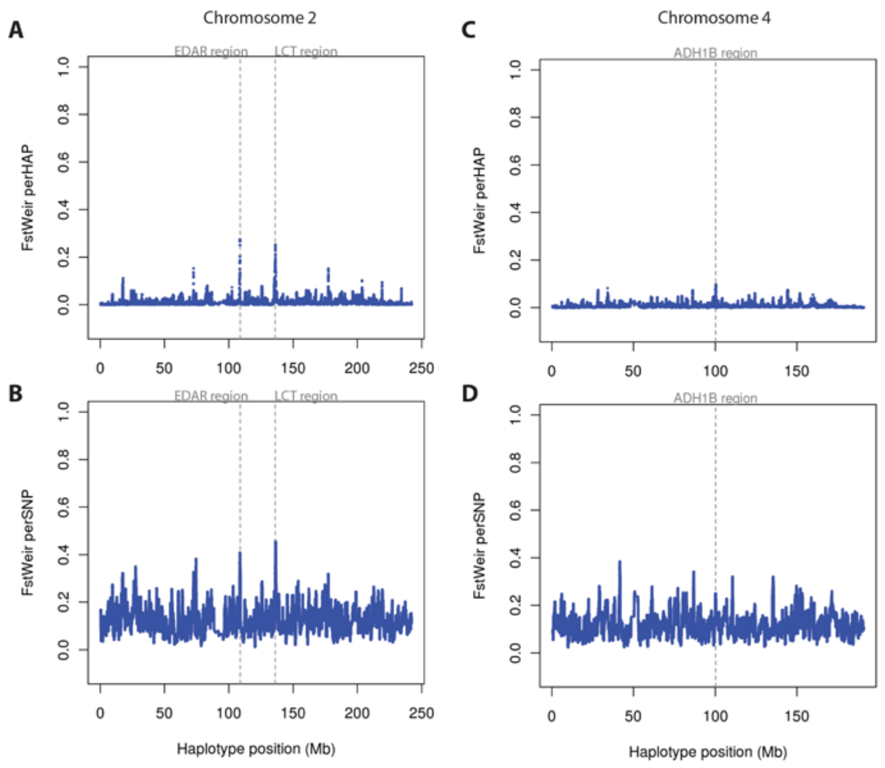

Figure S9: Mean of the absolute iHS value calculated in windows with 200 SNPs on HapMap III phased data. A 1 SNP step length between windows was utilized. The genomic positions of the genes LCT, EDAR (both on chromosome 2) and ADH1B (on chromosome 4) are indicated. A to C: chromosome 2, D to F: chromosome 4.

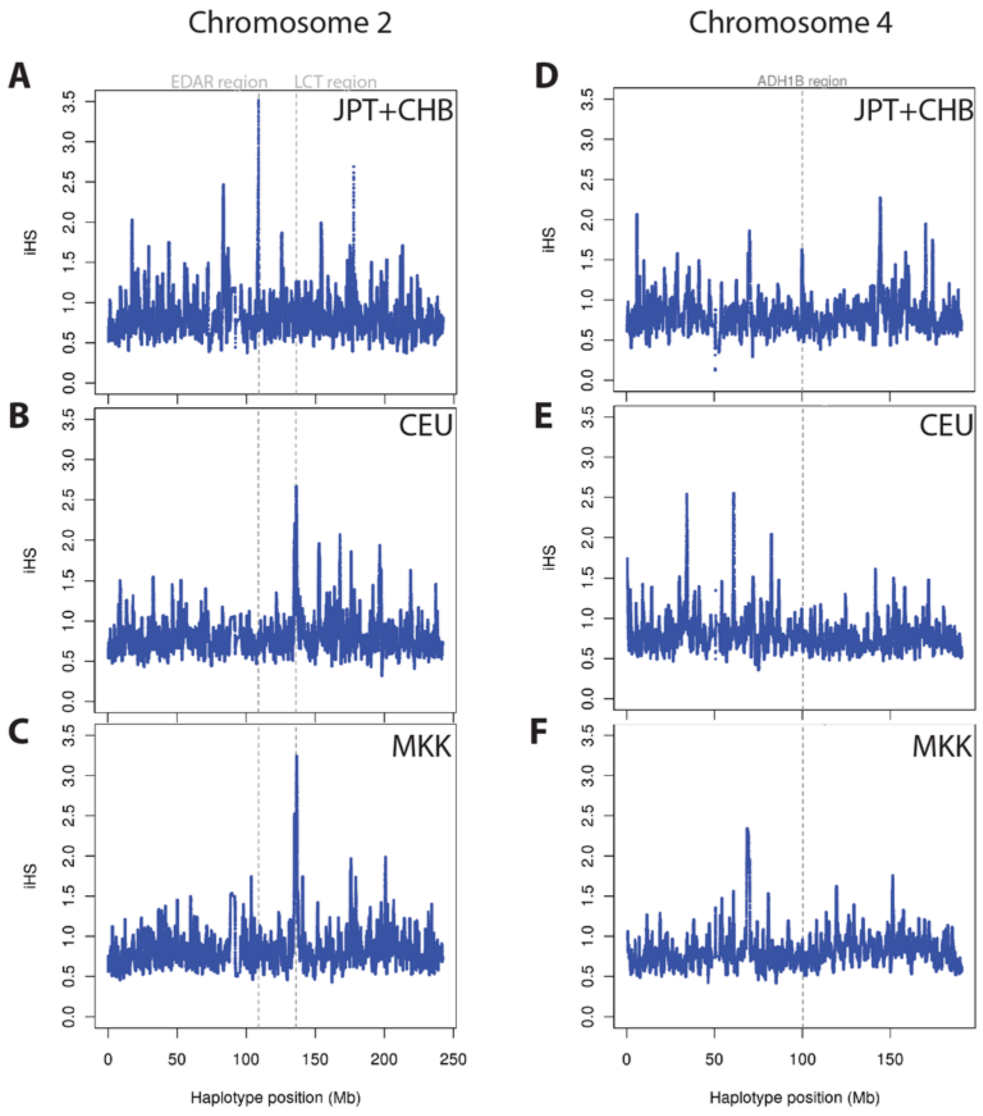

Figure S10: Mean XP-EHH calculated in windows with 200 SNPs on HapMap III phased data. A 1 SNP step length between windows was utilized. The genomic positions of the genes LCT, EDAR (both on chromosome 2) and ADH1B (on chromosome 4) are indicated. XP-EHH is calculated in one population (with 17 individuals) compared to the merged two others (34 individuals).

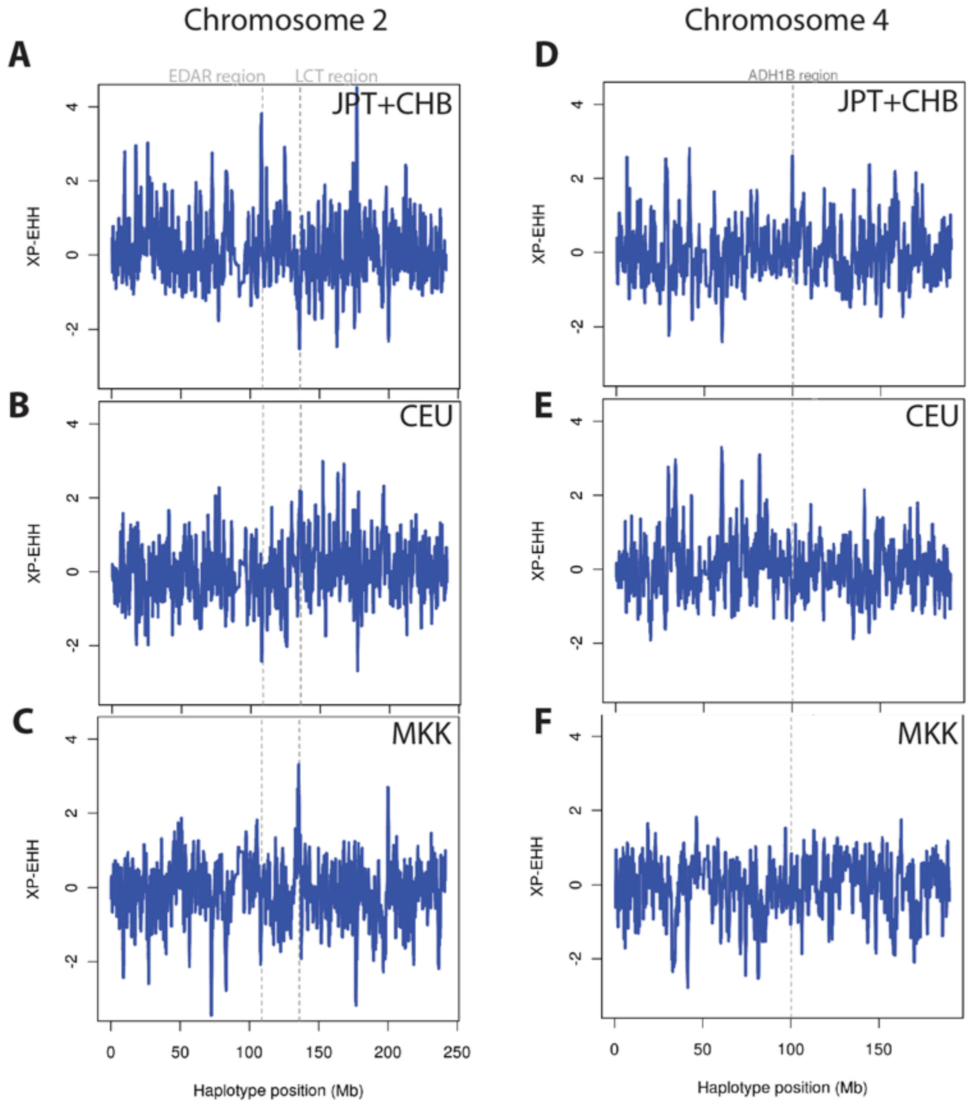

Figure S11: Mean value of the different statistics calculated in windows with 200 SNPs on HapMap III phased data. A 1 SNP step length between windows was utilized. Zoom-in around the LCT-gene region. F_{ST} based on the three populations CEU, JPT+CHB, MKK (all down sampled to 17 individuals) while MFPH and XP-EHH are calculated in one population (with 17 individuals) compared to the merged two others (34 individuals). **A:** in CEU **B:** in MKK.

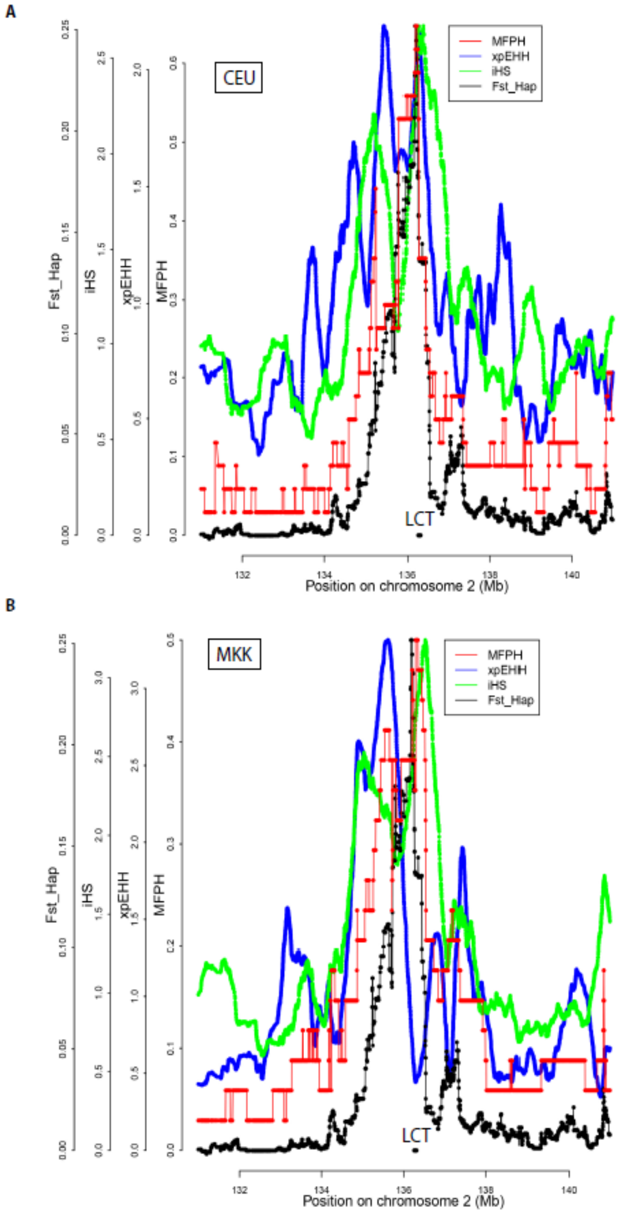

Figure S12: Mean value of the different statistics calculated in windows with 200 SNPs on HapMap III phased data. A 1 SNP step length between windows was utilized. Zoom-in around ADH1B and EDAR in JPT+CHB. F_{ST} based on the three populations CEU, JPT+CHB, MKK (all down-sampled to 17 individuals) while MFPH and XP-EHH are calculated in JPT+CHB (with 17 individuals) compared to the merged CEU and MKK (34 individuals). **A:** the ADH1B-gene region on chromosome 4, **B:** the EDAR-gene region on chromosome 2.

A

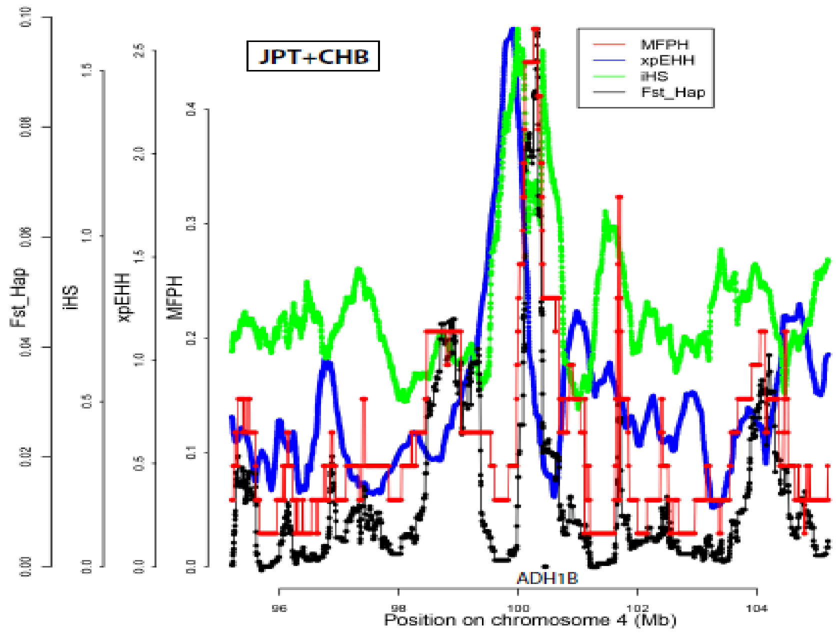

B

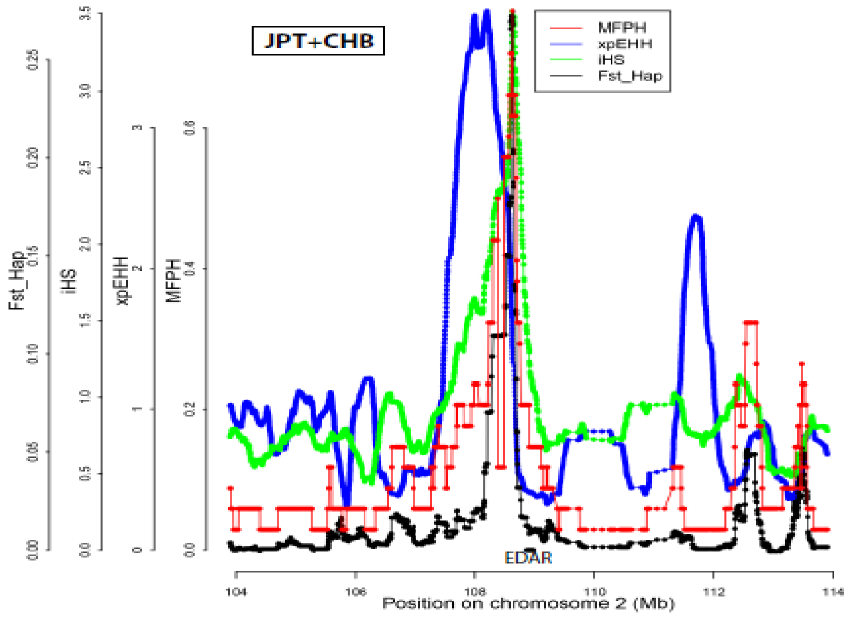

Figure S13: MFPH on HapMap III phased data for chromosome 2 taking all populations into account. A-C is replicated from figure 5 in the main text for comparison. Window-size is 200 SNPs with a 1 SNP step length between windows. The genomic positions of the genes LCT and EDAR are indicated. In D-F one focal population is compared to all Hapmap 3 populations (instead of only two groups as in A-C). Note that the sample size is lower (9 individuals) in D-F. The selection signal around the LCT gene is lost in CEU and the selection signal around the EDAR gene is lost in the JPT+CHB populations.

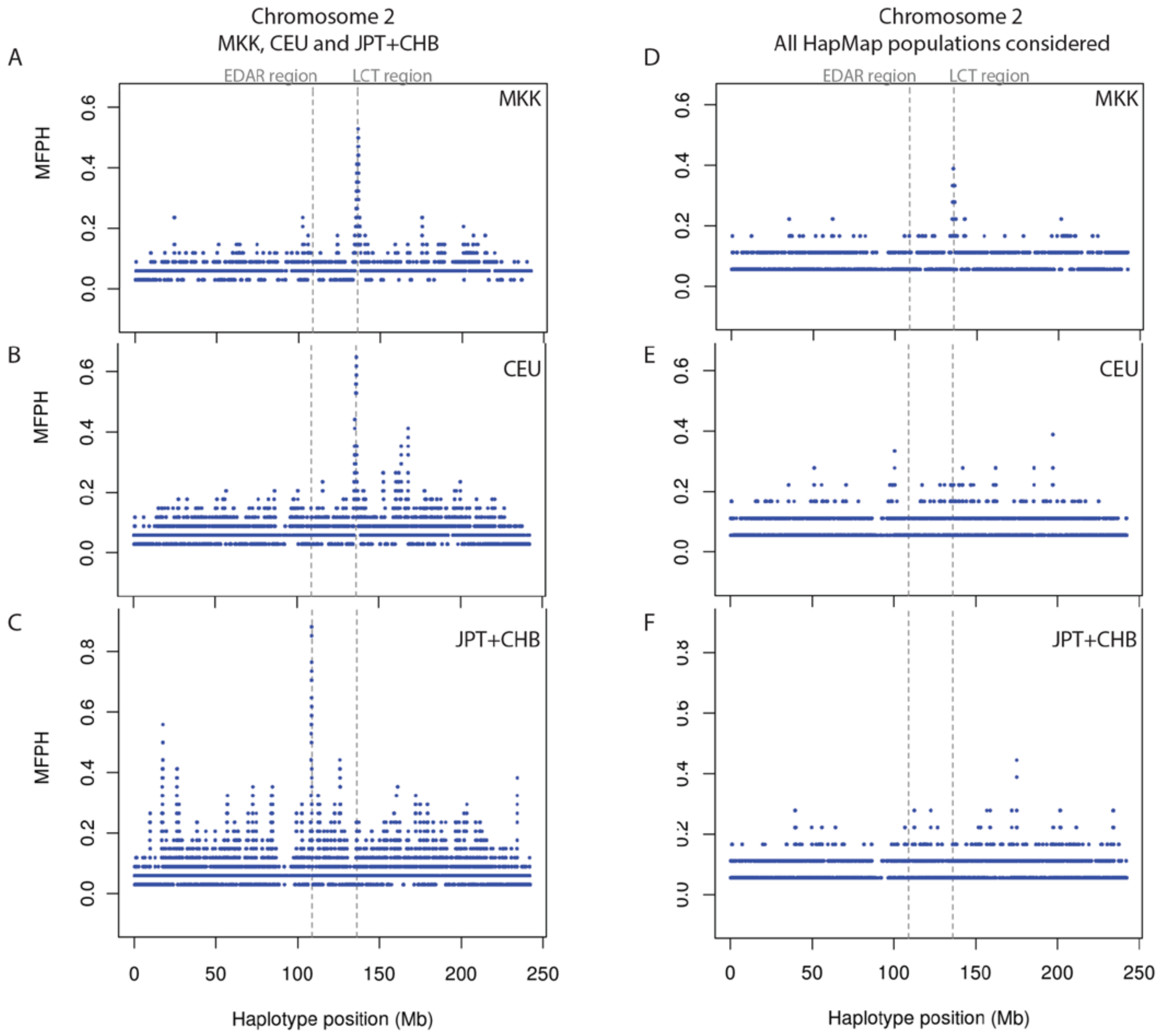

Figure S14: The top MFPH windows with XP-EHH, iHS and F_{ST} SNP after excluding chromosome 2 in MKK. For XP-EHH, the reference population was the combined CEU and JPT+CHB. F_{ST} values were calculated for each SNP using all three populations and with negative values set to zero Transparent points are values less than two standard deviations from the genome-wide mean. Non-transparent dots are values that are more than two standard deviation from the mean. Horizontal dotted bars give the genome-wide mean value of the statistics (standard deviation in dotted vertical bars) while continuous bars show the local mean. Horizontal bars in yellow show the gene positions. **A**) Region around 51mb on chromosome 3 **B**) Region around 101mb on chromosome 3.

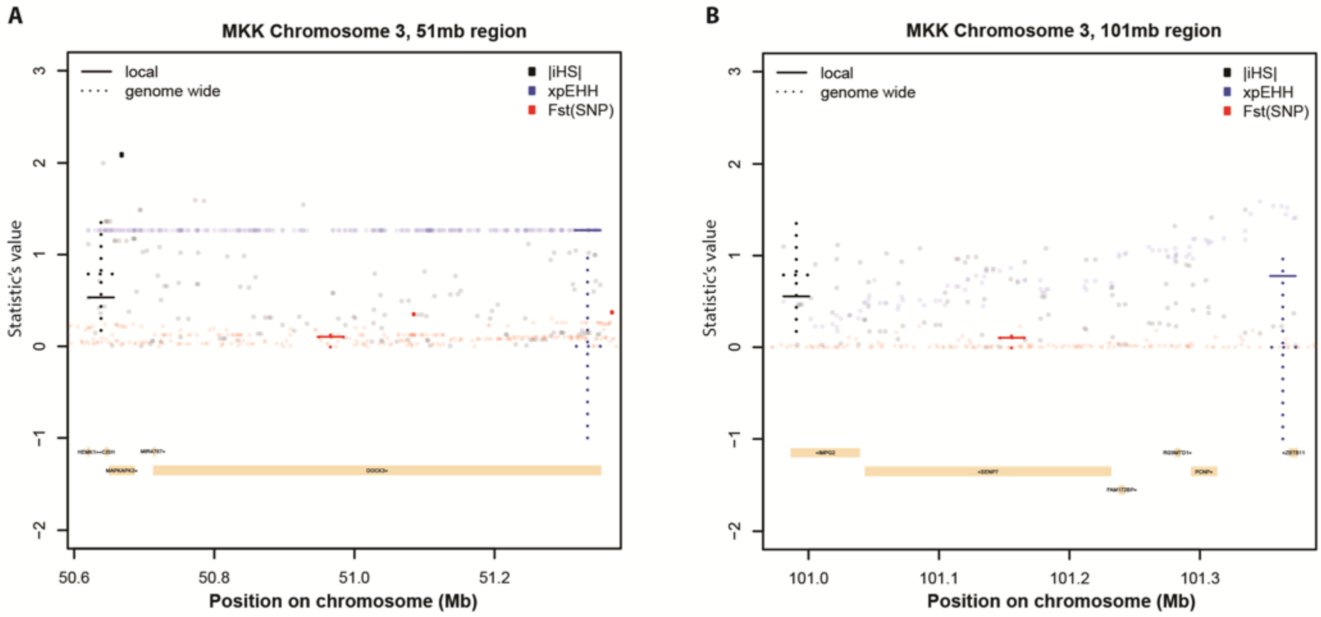

Figure S15: The top MFPH windows with XP-EHH, iHS and F_{ST} SNP after excluding chromosome 2 in CEU. For XP-EHH, the reference population was the combined MKK and JPT+CHB. F_{ST} values were calculated for each SNP using all three populations and with negative values set to zero. Transparent points are values less than two standard deviations from the genome-wide mean. Non-transparent points are values that are more than two standard deviation from the mean. Horizontal dotted bars give the genome-wide mean value of the statistics (standard deviation in dotted vertical bars) while continuous bars show the local mean. Horizontal bars in yellow show the gene positions.

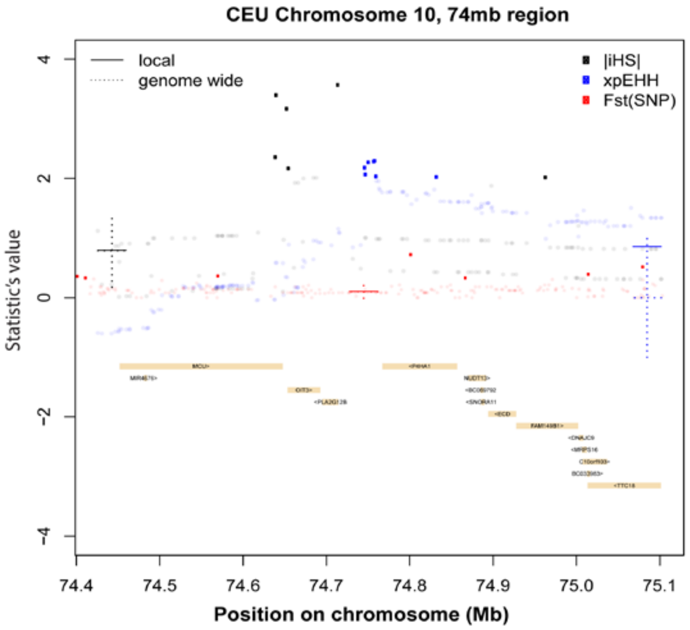

Figure S16: Visual genotypes of SNPs in the 51mb region of chromosome 3 covering MAPKAPK3, CISH and DOCK3 genes in three pygmy populations (Baka, Bakola, Bedzan; (Lachance *et al.*, 2012) and in MKK (Drmanac *et al.*, 2010). The region contains a total of 1619 SNPs, we show here SNPs with a minimum allele frequency of 30% (312 SNPs). Each line represents a SNP and each column an individual. 1: 50.6mb to 51mb, 2: 51mb to 51.4 mb. MKK_4 is different from the 3 other MKK individuals and Bedzan_1 is different from the 4 other pygmy individuals. The latter is likely to be a consequence of the Bedzan population being the most admixed pygmy population (Verdu *et al.*, 2009).

Individual references in the databases (Assembly Pipeline version 1.10 and CGA Tools 1.4):

- Baka_1: GS00319-DNA_A01_1100_37-ASM
- Baka_2: GS00319-DNA_B01_1100_37-ASM
- Baka_3: GS00319-DNA_C01_1100_37-ASM
- Bakola_1: GS00319-DNA_D01_1100_37-ASM
- Bedzan_1: GS00319-DNA_E01_1100_37-ASM
- MKK_1: NA21732-200-37-ASM
- MKK_2: NA21733-200-37-ASM
- MKK_3: NA21737-200-37-ASM
- MKK_4: NA21767-200-37-ASM

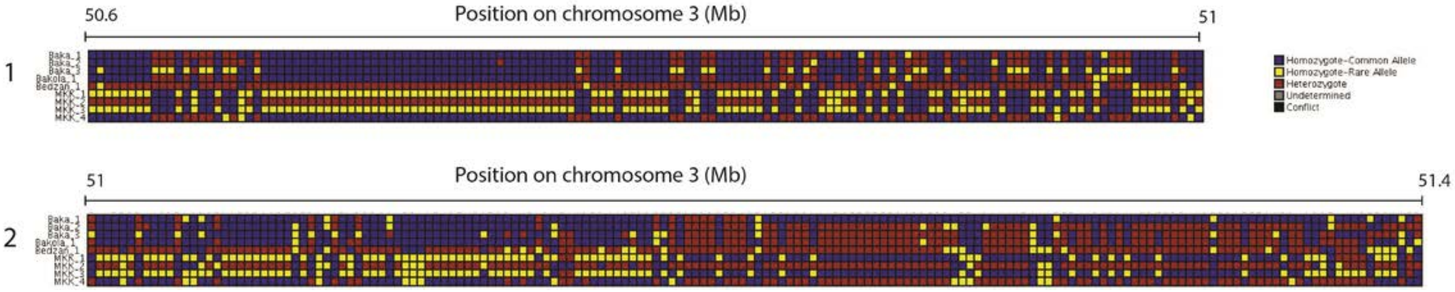

Table S1: The parameters and their values used in the simulations.

	Parameter	Symbol	Unit	Value
Population Parameters	Population size	N	Number of diploid individuals in a population	500
	Sampling size	n	Sampled individuals per population	15
	Sequence length	L	Base-pair (kb)	100
	Migration rate	m	Individual/population/generation	1-30
Genetic Parameters	Mutation rate	$\theta = 4N\mu$	Mutation event/site/generation	0.001
	Recombination rate	$\rho = 4Nr$	Recombination event/site/generation	0.001-0.02
Selection	Population-scaled Selection coefficient	$G = 2Ns$	Selective effect*population	0-500
Time Parameters	Burn-in Time	$B = 5PN$	Generations	5000
	Mutation Time	t_m	Generations after the split	100
	Sampling Time	t_s	Generations after t_m	1-1500
Analysis Parameters	Window	w	Base-pair (kb)	1, 5, 10
	Step	$step$	Base-pair (kb)	0.5, 2.5, 5

Table S2: Frequency of the most frequent allele defined in a window of size 5 kb surrounding site 50,001 in population 3. The first ("Total") to the fifth column ("f(non-private)") refers to simulations (among a 100 simulations) for which the value of MFPH was at least two standard deviations higher than the mean (mean and standard deviation were calculated on neutral simulations with G=0,ρ=0.001, m=1, θ=0.001, t_s=50, t_m=100, N=500). The sixth to the tenth columns refers to simulations were the value of MFPH was less than two standard deviations higher than the mean. "Total" is the total number of simulations. "Private" is the number of simulations (among the "total") where the most frequent haplotype only existed in population 3, "f(private)" is the mean (population) frequency of these haplotypes. "Non-private" is the number of simulations (among the "total") where the most frequent haplotype in population 3 existed in at least one of population 1 or 2 and "f(non-private)" is the mean frequency of these haplotypes in population 3. Note that "unique" is used instead of "private" since, in the main text, the latter was defined relative to a sample while "unique" is relative to the whole (sub)population.

Simulation Type		SIGNIFICANT					NON-SIGNIFICANT				
		Total	Private	f(private)	Non private	f(non private)	Total	Private	f(private)	Non private	f(non private)
Migration	5	35	33	16.03	2	14.00	65	3	6.00	62	16.66
	10	25	21	13.19	4	13.75	75	1	3.00	74	16.42
	20	10	9	11.89	1	14.00	90	0	x	90	13.30
	30	19	11	7.55	8	9,375	81	0	x	81	11.95
Selection	10	7	6	10.83	1	6.00	93	31	5.90	62	8.24
	50	11	9	10.11	2	6.50	89	25	5.64	64	8.41
	100	36	36	14.25	0	x	64	22	6.18	42	9.33
	200	73	73	23.73	0	x	27	0	x	27	24.04
	250	69	67	25.39	2	17.00	31	0	x	31	25.35
	300	58	58	25.19	0	x	42	0	x	42	26.02
	500	50	48	25.94	2	16.50	50	0	x	50	26.50
Sampling time	10	17	8	9.50	9	8.44	83	15	5.20	68	8.78
	100	40	40	22.50	0	x	60	0	x	60	22.39
	150	30	30	18.30	0	x	70	1	10.00	69	20.22
	200	26	26	19.69	0	x	74	6	7.33	68	18.31
	400	13	13	14.46	0	x	87	21	7.43	66	14.17
	600	5	5	15.40	0	x	95	46	7.72	49	11.80
	1000	15	15	11.40	0	x	85	47	6.87	38	10.92
	1500	7	7	13.57	0	x	93	52	7.02	41	8.90
Rho	1	73	71	18.77	2	8.00	27	6	8.00	21	19.14
	5	68	67	14.96	1	11.00	32	11	4.91	21	16.00
	10	73	72	13.13	1	15.00	27	16	5.06	11	12.00
	20	64	63	10.27	1	7.00	36	28	3.96	8	10.25

Table S3: Number of simulated cases out of a 100 that show a significant MFPH value (greater than the mean of the neutral case plus 2sd). "Rephased only" refers to the number of cases that were significant only in the rephased data, "Known phase only" refers to the number of cases that were significant only in the original known phase.

	rho	Rephased only	Found in both	Known phase only
Neutral	0.001	2	0	0
Neutral	0.005	0	4	2
Neutral	0.01	4	2	0
Neutral	0.02	3	1	3
Selected	0.001	9	**60**	5
Selected	0.005	9	**58**	7
Selected	0.01	10	**66**	4
Selected	0.02	9	**52**	9

Table S4: The correlation between MFPH with MKK as focal population and the size of windows in cM based on the genetic map from MKK (d_{mkk}), the genetic map from CEU (d_{ceu}), the difference between these (d_{mkk}-d_{ceu}), and the scaled difference between these ((d_{mkk}-d_{ceu})/(0.5(d_{mkk}+d_{ceu}))). Numbers outside parentheses are Pearson correlations and numbers within parentheses are Spearman correlations. 'all' represent genome wide correlations.

chr	$\frac{d_{mkk}-d_{ceu}}{0.5(d_{mkk}+d_{ceu})}$	$d_{mkk}-d_{ceu}$	d_{mkk}	d_{ceu}
1	0.14 (0.14)	0.1 (0.12)	-0.35 (-0.36)	-0.36 (-0.37)
2	0.04 (0.06)	0.04 (0.04)	-0.31 (-0.41)	-0.3 (-0.39)
3	0.08 (0.11)	0.04 (0.06)	-0.39 (-0.46)	-0.38 (-0.45)
4	0.11 (0.12)	0.04 (0.05)	-0.41 (-0.41)	-0.42 (-0.42)
5	0.12 (0.14)	0.08 (0.1)	-0.38 (-0.48)	-0.37 (-0.47)
6	0.16 (0.18)	0.1 (0.09)	-0.47 (-0.52)	-0.44 (-0.5)
7	0.12 (0.18)	0.16 (0.16)	-0.22 (-0.23)	-0.3 (-0.29)
8	0.08 (0.1)	-0.03 (-0.01)	-0.51 (-0.55)	-0.47 (-0.53)
9	-0.04 (-0.03)	-0.1 (-0.1)	-0.42 (-0.46)	-0.36 (-0.39)
10	0.11 (0.06)	-0.0 (-0.02)	-0.38 (-0.42)	-0.34 (-0.39)
11	0.14 (0.14)	0.08 (0.11)	-0.44 (-0.51)	-0.47 (-0.51)
12	0.07 (0.05)	0.01 (0.0)	-0.43 (-0.48)	-0.41 (-0.47)
13	0.1 (0.12)	0.07 (0.08)	-0.38 (-0.41)	-0.36 (-0.4)
14	-0.01 (0.03)	-0.04 (-0.03)	-0.39 (-0.48)	-0.36 (-0.45)
15	0.13 (0.12)	0.01 (0.02)	-0.54 (-0.62)	-0.52 (-0.59)
16	-0.14 (-0.09)	-0.02 (-0.02)	-0.5 (-0.51)	-0.46 (-0.48)
17	0.04 (0.08)	0.08 (0.09)	-0.42 (-0.43)	-0.37 (-0.4)
18	0.12 (0.13)	0.08 (0.1)	-0.4 (-0.42)	-0.42 (-0.45)
19	0.05 (0.0)	0.05 (0.02)	-0.35 (-0.34)	-0.35 (-0.32)
20	0.1 (0.13)	0.09 (0.13)	-0.3 (-0.3)	-0.3 (-0.29)
21	0.11 (0.11)	0.08 (0.09)	-0.29 (-0.35)	-0.3 (-0.32)
22	-0.01 (-0.03)	0.04 (-0.02)	-0.4 (-0.42)	-0.38 (-0.39)
**all**	**0.08 (0.09)**	**0.04 (0.04)**	**-0.38 (-0.45)**	**-0.37 (-0.43)**

Table S5: The correlation between MFPH with CEU as focal population and the size of windows in cM based on the genetic map from CEU (d_{ceu}), the genetic map from MKK (d_{mkk}), the difference between these (d_{ceu}-d_{mkk}), and the scaled difference between these ($(d_{ceu}-d_{mkk})/(0.5(d_{ceu}+d_{mkk}))$). Numbers outside parentheses are Pearson correlations and numbers within parentheses are Spearman correlations. 'all' represent genome wide correlations.

chr	$\frac{d_{ceu}-d_{mkk}}{0.5(d_{ceu}+d_{mkk})}$	$d_{ceu}-d_{mkk}$	d_{ceu}	d_{mkk}
1	-0.24 (-0.27)	-0.19 (-0.26)	-0.56 (-0.7)	-0.54 (-0.67)
2	-0.09 (-0.18)	-0.11 (-0.18)	-0.5 (-0.71)	-0.51 (-0.71)
3	-0.21 (-0.22)	-0.11 (-0.14)	-0.59 (-0.74)	-0.59 (-0.75)
4	-0.2 (-0.21)	-0.07 (-0.1)	-0.52 (-0.67)	-0.51 (-0.67)
5	-0.26 (-0.28)	-0.16 (-0.23)	-0.59 (-0.74)	-0.59 (-0.74)
6	-0.3 (-0.34)	-0.19 (-0.23)	-0.58 (-0.73)	-0.58 (-0.7)
7	-0.17 (-0.21)	-0.07 (-0.09)	-0.54 (-0.67)	-0.53 (-0.64)
8	-0.2 (-0.23)	-0.06 (-0.09)	-0.58 (-0.73)	-0.59 (-0.71)
9	-0.19 (-0.19)	-0.06 (-0.06)	-0.57 (-0.71)	-0.56 (-0.68)
10	-0.21 (-0.24)	-0.11 (-0.15)	-0.53 (-0.65)	-0.53 (-0.63)
11	-0.2 (-0.17)	-0.08 (-0.13)	-0.58 (-0.72)	-0.56 (-0.72)
12	-0.19 (-0.21)	-0.1 (-0.14)	-0.5 (-0.71)	-0.48 (-0.69)
13	-0.23 (-0.24)	-0.15 (-0.21)	-0.59 (-0.72)	-0.59 (-0.71)
14	-0.19 (-0.18)	-0.09 (-0.11)	-0.57 (-0.72)	-0.56 (-0.73)
15	-0.15 (-0.25)	-0.04 (-0.12)	-0.54 (-0.73)	-0.55 (-0.72)
16	0.02 (-0.06)	-0.1 (-0.16)	-0.59 (-0.72)	-0.58 (-0.72)
17	-0.25 (-0.33)	-0.23 (-0.32)	-0.57 (-0.77)	-0.58 (-0.75)
18	-0.14 (-0.14)	-0.08 (-0.09)	-0.52 (-0.63)	-0.49 (-0.6)
19	-0.27 (-0.2)	-0.16 (-0.18)	-0.51 (-0.6)	-0.48 (-0.58)
20	-0.27 (-0.29)	-0.25 (-0.35)	-0.6 (-0.71)	-0.57 (-0.67)
21	-0.22 (-0.25)	-0.11 (-0.16)	-0.55 (-0.67)	-0.56 (-0.71)
22	-0.24 (-0.2)	-0.11 (-0.13)	-0.54 (-0.71)	-0.56 (-0.69)
**all**	**-0.19 (-0.22)**	**-0.11 (-0.16)**	**-0.54 (-0.72)**	**-0.54 (-0.71)**

Supplementary references:

Drmanac R, Sparks AB, Callow MJ, Halpern AL, Burns NL, Kermani BG, Carnevali P, Nazarenko I, Nilsen GB, Yeung G, Dahl F, Fernandez A, Staker B, Pant KP, Baccash J, Borcherding AP, Brownley A, Cedeno R, Chen L, Chernikoff D, Cheung A, Chirita R, Curson B, Ebert JC, Hacker CR, Hartlage R, Hauser B, Huang S, Jiang Y, Karpinchyk V, et al.: **Human Genome Sequencing Using Unchained Base Reads on Self-Assembling DNA Nanoarrays**. *Science* 2010, **327**:78–81.

Lachance J, Vernot B, Elbers CC, Ferwerda B, Froment A, Bodo J-M, Lema G, Fu W, Nyambo TB, Rebbeck TR, Zhang K, Akey JM, Tishkoff SA: **Evolutionary history and adaptation from high-coverage whole-genome sequences of diverse African hunter-gatherers**. *Cell* 2012, **150**:457–469.

Scheet, P and Stephens, M: **A fast and flexible statistical model for large-scale population genotype data: applications to inferring missing genotypes and haplotypic phase**. *Am J Hum Genet* 2006*, 78:629–644*

Verdu P, Austerlitz F, Estoup A, Vitalis R, Georges M, Théry S, Froment A, Le Bomin S, Gessain A, Hombert JM, Van der Veen L, Quintana-Murci L, Bahuchet S, Heyer E: **Origins and Genetic Diversity of Pygmy Hunter-Gatherers from Western Central Africa.** *Current Biology* 2009, **19**: 312-318.

Weir BS: *Genetic Data Analysis 2*. Sinauer Associates; 1996.
